# In Vitro and In Silico Potential Inhibitory Effects of New Biflavonoids from *Ochna rhizomatosa* on HIV-1 Integrase and *Plasmodium falciparum*

**DOI:** 10.3390/pharmaceutics14081701

**Published:** 2022-08-15

**Authors:** Angélique Nicolas Messi, Susan Lucia Bonnet, Brice Ayissi Owona, Anke Wilhelm, Eutrophe Le Doux Kamto, Joseph Thierry Ndongo, Xavier Siwe-Noundou, Madan Poka, Patrick H. Demana, Rui W. M. Krause, Joséphine Ngo Mbing, Dieudonné Emmanuel Pegnyemb, Christian G. Bochet

**Affiliations:** 1 Department of Organic Chemistry, Faculty of Science, University of Yaounde I, Yaounde P.O. Box 812, Cameroon; 2 Department of Chemistry, University of the Free State, 205 Nelson Mandela Avenue, Bloemfontein 9301, South Africa; 3 Department of Chemistry, University of Fribourg, Chemin du Musée 9, CH-1700 Fribourg, Switzerland; 4 Department of Biochemistry, Faculty of Science, University of Yaounde I, Yaounde P.O. Box 812, Cameroon; 5 Department of Chemistry, Higher Teacher Training College, University of Yaounde 1, Yaounde P.O. Box 47, Cameroon; 6 Department of Pharmaceutical Sciences, School of Pharmacy, Sefako Makgatho Health Sciences University, Pretoria 0204, South Africa; 7 Nanomaterials and Medicinal Organic Chemistry Laboratory, Department of Chemistry, Rhodes University, Grahamstown 6140, South Africa

**Keywords:** *Ochna rhizomatosa*, biflavonoids, HIV-1 replication, *Plasmodium falciparum* NF*54*, structure–activity relationships, molecular docking

## Abstract

The aim of this study was to identify bioactive secondary metabolites from *Ochna rhizomatosa* with potential inhibitory effects against HIV and *Plasmodium falciparum. A* phytochemical study of *O. rhizomatosa* root barks resulted in the identification of three new biflavonoids (**1**–**3**), along with four known ones (**4**–**7**). Compound **7** (Gerontoisoflavone A) was a single flavonoid present in the rootbark of the plant and was used as a reference. Compound **1** (IC_50_ = 0.047 µM) was the only one with a noteworthy inhibitory effect against HIV-1 integrase in vitro. Chicoric acid (IC_50_ = 0.006 µM), a pure competitive inhibitor of HIV-1 integrase, was used as control. Compound **2** exhibited the highest antiplasmodial activity (IC_50_ = 4.60 µM) against the chloroquine-sensitive strain of *Plasmodium falciparum* NF*54*. Computational molecular docking revealed that compounds **1** and **2** had the highest binding score (−121.8 and −131.88 Kcal/mol, respectively) in comparison to chicoric acid and Dolutegravir (−116 and −100 Kcal/mol, respectively), towards integrase receptor (PDB:3LPT). As far as Plasmodium-6 cysteine s48/45 domain inhibition is concerned, compounds **1** and **2** showed the highest binding scores in comparison to chloroquine, urging the analysis of these compounds in vivo for disease treatment. These results confirm the potential inhibitory effect of compounds **1** and **2** for HIV and malaria treatment. Therefore, our future investigation to find inhibitors of these receptors in vivo could be an effective strategy for developing new drugs.

## 1. Introduction

Malaria and HIV/AIDS are among the main illnesses Sub-Saharan Africa is facing. In many countries in this area, such as Cameroon, both diseases are endemic, and the HIV/AIDS infections may increase the burden of malaria. This may occur by increasing the susceptibility to malaria infection, hence, making these diseases deadlier [1].

Malaria is responsible for over one million deaths annually, of which more than 92% occur in Africa. Indeed, malaria continues to be the leading cause of child mortality in developing countries [2]. According to data from Cameroon’s Ministry of Public Health, malaria was recorded as the leading cause of death in Cameroon, reporting over 3000 deaths in 2018. Malaria affects about two million Cameroonians each year, and mortality due to this disease is higher among children under five years and pregnant women [3]. The World Health Organization (WHO) currently recommends parental artesumate as the drug of choice in the treatment of severe malaria and quinine as the second-line drug in children and pregnant women [2,4]. However, the application of artemisinin-based combination therapies (ACTs) is not effective due to the fact that the malaria parasite has developed resistance to many of the currently available drugs, including the ACTs therapies [5]. Therefore, many other drugs are preferred by the population, including chloroquine.

HIV/AIDS has constituted a devastating global epidemic since the 1980s. In 2019, while more than 37.9 million people were carrying HIV, there was a high number of children, i.e., 1.7 million among those people, and roughly 770,000 people lost their lives due to AIDS-related illnesses around the world [6]. A staggering 25.8 million (70%) of those people are in Sub-Saharan Africa, which is the center of this epidemic. In Cameroon, 540,000 (3.6%) adults aged between 15 and 49 are living with HIV. AIDS represents the disease with the highest rate of death in the 15–50 age group, as around 18,000 related deaths were reported in 2019 [7]. Thus far, there is no effective drug that can treat HIV/AIDS, and the highly active antiretroviral therapy (HAART), essentially comprising nucleoside and non-nucleoside reverse transcriptase inhibitors as well as protease inhibitors, has been extensively used to reduce the proliferation of the HIV infection. Notwithstanding the valuable contribution of HAART towards the improvement of the life standard of people living with HIV/AIDS, their prolonged use is compromised as a result of the development of virus resistance, unavailability and the lack of a curative effect [8]. This, therefore, underscores the need to explore novel therapeutic strategies such as the incorporation of medicinal plants for the management and treatment of viral diseases and related opportunistic infections.

Many studies reported that flavonoids are effective in suppressing HIV replication [9]. Biflavonoids are a subclass of flavonoids and are naturally formed by two identical or nonidentical flavonoid units [10]. Their broad biological activity, such as antiplasmodial effects, has sparked the interest of many researchers [11,12]. Moreover, the bioactivities of biflavonoids are stronger than those of flavonoids [13]. Biflavonoids are naturally split into three linkage types: C–C type [14], C–O–C type [13] and a connected C–C/C–O–C type [15]. Within the C–C connection type, most of the biflavonoids are made using an interflavonoid interconnection between C-6/8 and C-3′, C-2′, C-3, C-6 or C-8 [16].

Nonetheless, the phytochemical investigation of *Ochna rhizomatosa,* as well as the assessment of the inhibitory effects on HIV-1 integrase replication enzyme of biflavonoids, have not been previously studied. Biflavonoids have advantages over monomeric flavonoids since biflavonoids are able to survive first-pass metabolism, which inactivates most flavonoids [13]. Unlike their monomeric constituents, the occurrence of biflavonoids in nature is restricted to some species, such as *Ochna* and *Campylospermum* [12].

*Ochna rhizomatosa* is a small tree that can grow up to 1 m tall and is mostly found in African woodlands. This plant species belongs to the Ochnaceae family, from which a large number of flavonoids, biflavonoids and chalcones have been isolated [17,18,19]. Recently, we isolated two new biflavonoids and six known ones from *O. schweinfurthiana.* Some of the isolates were evaluated for their antiplasmodial and antioxidant activities [12].

In this study, the chemical constituents of the CH_2_Cl_2_/MeOH; (1:1) extract of the root bark of *O. rhizomatosa* were investigated in order to find a potential inhibitor of HIV-1-integrase and antiplasmodial lead compounds. As a result, three new uncommon C–C type biflavonoids formed through rare types of Ca_1_–Ca_2_ and C_1″_–Cb_2_ bonds (**1**–**3**) are described here for the first time along with four known compounds (**4**–**7**). Their structures were elucidated by means of their UV, IR, NMR, HRESIMS, and CD spectroscopic data analyses. The absolute configuration at (Ca_2_) of the new biflavonoids was established for the first time. Structure-based-drug design has long been recognized as a promising tool to discover drug inhibitors and has become a famous strategy for discovering new lead compounds for drug development. Taking advantage of molecular docking tools, the binding potential of the isolates was investigated on integrase and *Plasmodium* receptors, which are key enzymes involved in HIV and *Plasmodium* invasion of human cells.

## 2. Materials and Methods

### 2.1. Plant Materials Collection

The root barks of *Ochna rhizomatosa* F. Hoffm (Ochnaceae) were collected from the Dang district, Adamaoua region, Cameroon (GPS data 7°34′30″ N 13°30′35″ E 1.84 km) in December 2019. The plant was identified at the National Herbarium of Cameroon, where a voucher specimen (No. 39120HNC) was deposited.

### 2.2. General Experimental Procedures

Optical rotations ([ᾳ]^20^_D_) were measured in methanol on a JASCO 810 polarimeter in a 1 cm tube. The FTIR spectra were recorded on a Perkin Elmer FT-IR spectrometer (Thermo Scientific, Madison, WI, USA). The NMR spectral data were recorded using a Bruker Avance DPX-400 spectrometer operating at the frequencies of 400 MHz (^1^H) and 100 MHz (^13^C). The samples were dissolved in MeOH-*d*_4_ prior to recording. Chemical shift values were given in parts per million (ppm) from the internal standard. HRESIMS spectral data resulted from a MSQ Thermo Finnigan. Column chromatography (CC) was carried out on a medium preparative liquid chromatography (MPLC) system (RP-18, 0.040–0.063 mm, Merck MGaA, Darmstadt, Germany), Sephadex LH-20 (0.025–0.100 mm; GE Healthcare, Danderyd, Sweden) using silica gel 60 (Merck, 0.040–0.063 mm Qingdao, China). Thin layer chromatography (TLC) experiments were performed on pre-coated Merck Kieselgel 60 F_254_ plates (20 × 20 cm^2^, 0.25 mm, Qingdao, China).

### 2.3. Extraction of Plant Materials and Isolation of Compounds

The root barks of *Ochna rhizomatosa* (500 g) were dried and crushed before extraction using CH_2_Cl_2_/MeOH 1:1 (3 × 3 L) at ambient temperature to afford 87 g of crude extract after evaporation under reduced pressure. Part of the crude extract (80 g) was chromatographed on silica gel using a stepwise gradient condition and a mixture of CH_2_Cl_2_/MeOH (100:0 to 5:1) as solvents, resulting in 185 fractions A–H. Fraction D (CH_2_Cl_2_/MeOH; 30:1) was further purified by column chromatography using Sephadex LH-20 in acetone to yield compounds **2** (16 mg) and **7** (30 mg). The purification of fraction E (CH_2_Cl_2_/MeOH; 20:1) by column chromatography was also performed using Sephadex LH-20 in the same conditions and afforded compounds **4** (15 mg) and **5** (20 mg). For fraction F (CH_2_Cl_2_/MeOH; 20:1) a purification via a conventional RP-C_18_ column eluting with an MeOH/H_2_O system resulted in the isolation of compound **3** (100 mg). The fraction G was chromatographed by a RP-C_18_ column using the same conditions to yield compound **1** (150 mg). Lastly, fraction H [CH_2_Cl_2_/MeOH (5:1)] was subjected to repeated Sephadex LH-20 column chromatography eluted with an isocratic elution of CH_2_Cl_2_/MeOH (10:1), and this resulted in the isolation of compounds **1** (11 mg) and **6** (40 mg).

**(*R*)-*rhizomatobiflavonoid A* (1):** Yellow powder; [ᾳ]^20^_D_—28.6 (c 0.6, MeOH); FTIR (KBr) υ_max_ 3287, 1652, 1564 and 1506 cm^−1^; (+)-HR-ESIMS *m/z* 533.1196 [M + Na]^+^ (calc. for C_30_H_22_O_8_Na, 533.1046); ^1^H and ^13^C NMR spectral data, see Table 1.

**(*R*)-*rhizomatobiflavonoid B* (2):** Yellow powder; [ᾳ]^20^_D_—2.2 (c 0.5, MeOH); (+)-HR-ESIMS *m/z* 567.1274 [M + H]^+^ (calc. for C_34_H_30_O_8_, 567.3501); ^1^H and ^13^C NMR spectral data, see Table 1.

**(*R*)-*rhizomatobiflavonoid C* (3):** Yellowish amorphous powder; [ᾳ]^20^_D_—1.8 (c 0.6, MeOH); (−)-ESIMS *m/z* 537.3 [M-H]^−^ (calc. for C_32_H_26_O_8_, 537.1); ^1^H and ^13^C NMR spectral data, see Table 1.

### 2.4. Biological Activities

#### 2.4.1. Evaluation of Anti-HIV Integrase Assay

The HIV-1 Integrase strand transfer inhibition assay was adapted from previously described methods [20,21]. Briefly, 20 nM double-stranded biotinylated donor DNA (5′-5-BiotinTEG/ACCCTTTTAGTCAGTGTGGAAAATCTCTAGCA-3′) annealed to (5′-ACTGCTAGAGATTTTCCACACTGACTAAAAG-3′) was immobilized in wells of streptavidin-coated 96 well microtiter plates (R&D Systems, Minneapolis, MN, USA). Following incubation at room temperature for 40 min and a stringent wash step, 5 µg/mL purified recombinant HIV-1 subtype CIN in integrase buffer 1 (50 µM NaCl, 25 µM Hepes, 25 µM MnCl_2_, 5 µM β-mercap to ethanol, 50 µg/mL BSA, pH 7.5) was added to individual wells. Test compounds and chicoric acid were added to individual wells to a final concentration of 20 µM. Recombinant HIV-1 subtype CIN was assembled on to the pre-processed donor DNA through incubation for 45 min at room temperature. Strand transfer reaction was initiated through the addition of 10 µM (final concentration) double-stranded FITC-labelled target DNA (5′-TGACCAAGGGCTAATTCACT/36-FAM/-3′) annealed to (5′-AGTGAATTAGCCCTTGGTCA-/36-FAM/-3′) in integrase buffer 2 (same as buffer 1, except 25 µM MnCl_2_ was replaced with 2.5 µM MgCl_2_). After an incubation period of 60 min at 37 °C, the plates were washed using PBS containing 0.05% Tween 20 and 0.01% BSA, followed by the addition of peroxidase-conjugated sheep anti-FITC antibody (Thermo Scientific, Waltham, MA, USA) diluted 1:1000 in the same PBS buffer. Finally, the plates were washed and peroxidase substrate (Sure Blue Reserve™, KPL, Gaithersburg, MD, USA) was added to allow for detection at 620 nm using a Synergy MX (BioTek^®^) plate reader. Absorbance values were converted to % enzyme activity relative to the readings obtained from the control wells (enzyme without inhibitor).

#### 2.4.2. Evaluation of Antiplasmodial Assay

The assessment of antiplasmodial activity in vitro was evaluated against the erythrocytic stages of chloroquine-sensitive *Plasmodium falciparum* strain NF*54* using a ^3^H-hypoxanthine incorporation assay previously described [22], of which the chloroquine and pyrimethamine-resistant NF*54* strain originated from South Africa, and the standard drug chloroquine was obtained from Sigma-Aldrich (St. Louis, MO, USA). Compounds were dissolved in DMSO at 10 μg/mL and added to parasite cultures incubated in RPMI 1640 medium without hypoxanthine, supplemented with HEPES (5.94 g/L), NaHCO_3_ (2.1 g/L), neomycin (100 U/mL), Albumax, and washed human red cells A^+^ at 2.5% hematocrit (0.3% parasitemia). Serial sample dilutions of 113-fold dilution steps covering a range from 100 to 0.002 μg/mL were prepared. The 96-well plates were incubated in a humidified atmosphere at 37 °C; 4% CO_2_; 3% O_2_ and 93% N_2_. After 48 h, 50 μL of ^3^H-hypoxanthine (0.5 μCi) was added to each well.

#### 2.4.3. Molecular Docking

Ligand preparation: The 3D structures of compounds **1**, **2** and **7** were drawn using Molview software. Energy minimization was performed using the MM2 force field and saved as a MOL format. The missing charges and hybridization states of compound structures were assigned with the help of the MVD software.

Protein preparation: The 3D structures of different receptors were retrieved from the Protein Data Bank (PDB) (http://www.rcsb.org accessed on 22 July 2022). Integrase (PDB ID: 3LPT), and plasmodium falciparum key protein (PDB ID: 2LOE) were chosen respectively as targets. The proteins had one or two polypeptides and were co-crystallized with ligands. The targets were visually inspected, and reference ligands were identified for each receptor. Receptors were prepared for docking by removal of water molecules, ligands, cofactors and assigning bonds, bond order, hybridization and charges using the MVD software.

Docking search algorithm and scoring functions: MVD uses a Piecewise Linear Potential (PLP) algorithm as a scoring function for computational screening. In this study, the MolDock simplex evolution search algorithm was used for docking. Docking of compounds **1**, **2** and **7** in different receptors was performed, and the best poses generated were used based on the docking scores (expressed in Kcal/mol). Docking of compounds **3**, **4**, **5** and **6** were not pursued because no binding poses were obtained (data not shown). MVD software uses two scoring functions: the MolDock score and the ReRank score, where MolDock score is an E score (docking scoring function) defined as: **E score = E inter + E intra**. E inter: Sum of the ligand–protein interaction energy, ligand–water interaction energy and ligand–cofactor interaction energy [23]). E intra: internal energy of the ligand. ReRank score provides an estimation of the ligand–receptor interaction strength [24].

#### 2.4.4. Statistical Analysis

Data were represented as mean ± SD. Statistical analysis of the data was carried out using analysis of variance (ANOVA). Differences with *p*-value < 0.05 were considered statistically significant.

## 3. Results and Discussion

### 3.1. Identification of the Isolated Compounds 

**Figure 1:**
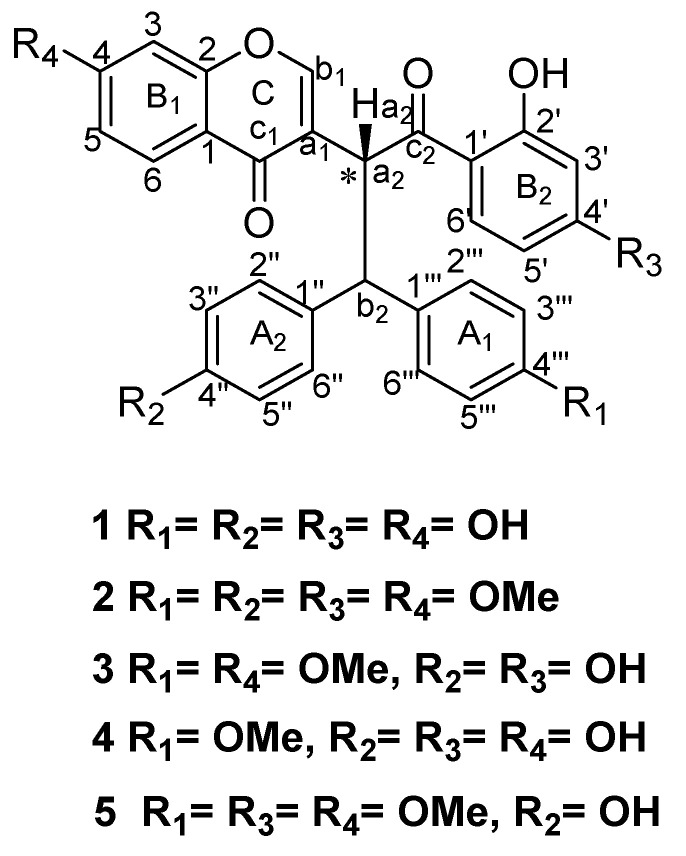

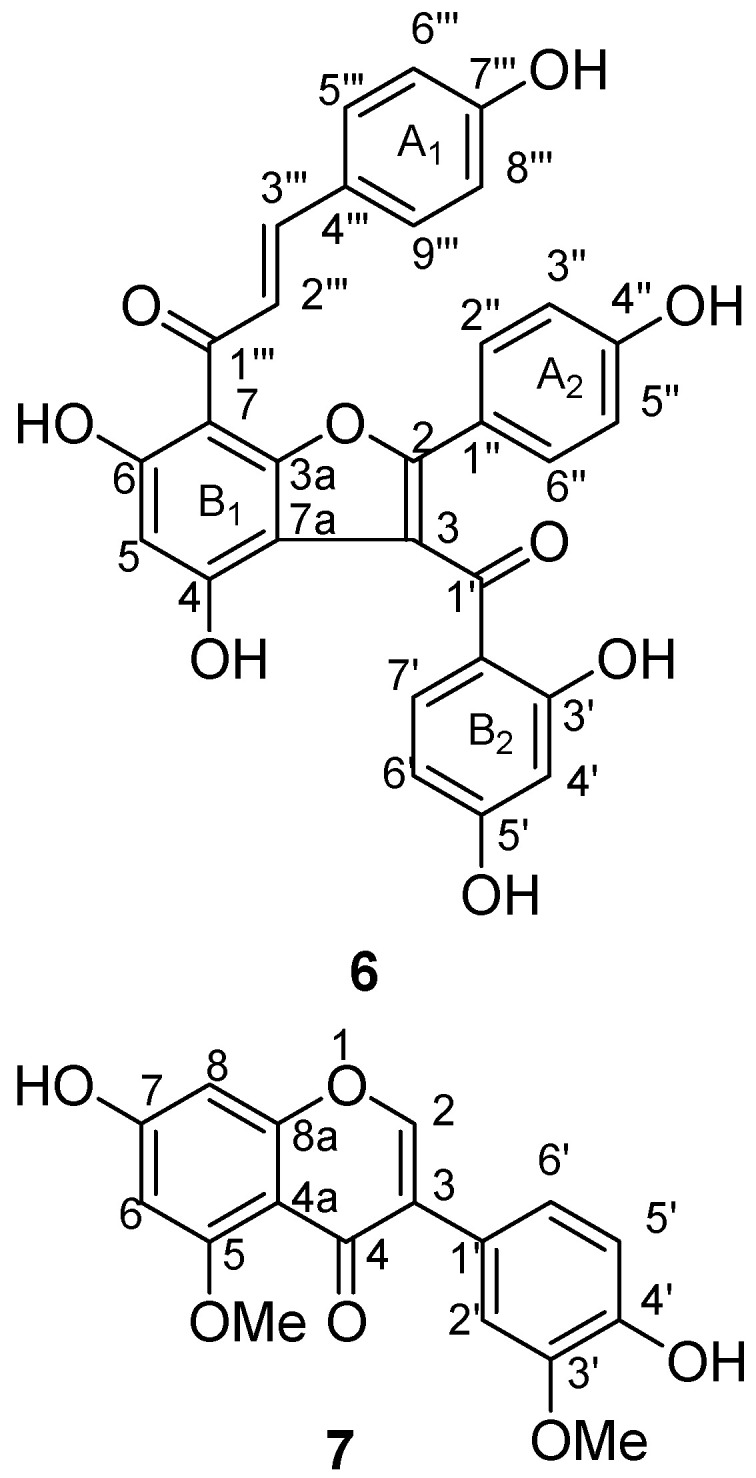
Chemical structures of the isolated compounds (**1**–**7**) from the root bark of *Ochna rhizomatosa*.

The crude (CH_2_Cl_2_/MeOH 1:1) extract obtained from *Ochna rhizomatosa* root barks was chromatographed using silica gel and Sephadex LH-20 columns to yield three new unusual biflavonoids consisting of an isoflavone fused with a dihydrochalcone skeleton (**1–3**), i.e., ^®^-rhizomatobiflavonoid A (**1**), (R)-rhizomatobiflavonoid B (**2**) a^®^(R)-rhizomatobiflavonoid C (**3**) (Figure 1) along with four known compounds, namely schweinfurthianone A (**4**) [12], shweinfurthianone B (**5**) [12], calodenine B (**6**) [12,25,26] and gerontoisoflavone A (**7**) (a single flavonoid present in the root barks of the plant and used as a reference) [12]. The chemical structures of the new compounds were identified by using their UV, IR, NMR, HRESIMS, and CD spectroscopic data analyses and optical rotation, whilst the known ones were elucidated by comparison with previously reported data.

Compound **1** was isolated as a yellow powder and gave a positive reaction with Neu’s reagent, indicative of biflavonoids [12]. The HR-ESIMS spectrum showed a sodium adduct ion peak at *m/z* 533.1196 [M + Na]^+^, which corresponds to the molecular mass of C_30_H_22_O_8_Na (calc. 533.1046 [M + Na]^+^). The spectral data from the UV analysis displayed absorptions at λ_max_ 243, 247 and 277 nm, suggestive of an isoflavonoid nucleus [27]. The IR spectrum disclosed vibration bands at υ_max_ 3287 (hydroxyl groups), 1652 (conjugated carbonyls) and between 1564 and 1506 cm^−1^, indicative of aromatic ring and conjugated double bonds, respectively.

The ^1^H NMR spectrum exhibited the characteristic signals of two aromatic AA′BB′-type proton systems at *δ*_H_ 7.17 (2H, d, *J* = 8.5 Hz, H-2‴/6‴) and 6.60 (2H, d, *J* = 8.5 Hz, H-3‴/5‴) for ring A_2_ and at *δ*_H_ 7.13 (2H, d, *J* = 8.5 Hz, H-2″/6″) and 6.65 (2H, d, *J* = 8.5 Hz, H-3″/5″) for ring A_1_ (see Figure S1). Two aromatic ABX-type proton signals resonating at *δ*_H_ 6.85 (1H, dd, *J* = 2.5, 9.0 Hz, H-5); 6.71 (1H, d, *J* = 2.5 Hz, H-3) and 7.88 (1H, d, *J* = 9.0 Hz, H-6) for ring B_1_ (1,2,4-trisubstituted ring) and also at *δ*_H_ 6.34 (1H, dd, *J* = 2.0, 9.0 Hz, H-5′); 6.14 (1H, d, *J* = 2.5 Hz, H-3′) and 8.15 (1H, d, *J* = 9.0 Hz, H-6′) for ring B_2_. One aliphatic AB spin system of two methine resonances is seen at *δ*_H_ 6.01 and 4.67 (d, *J* = 11.0 Hz, H-a_2_/b_2_). Moreover, the ^1^H NMR spectrum exhibited a singlet resonance at δ_H_ 8.23 (H-b_1_), characteristic of an unusual isoflavone [12,28].

The ^1^H-^1^H COSY spectrum allowed the establishment of the following spin-system sequences: H-2‴ to H-3‴, H-5‴ to H-6‴, H-2″ to H-3″, H-5″ to H-6″, H-5 to H-6, H-5′ to H-6′ and H-a_2_ to H-b_2_, which confirmed the presence of two aromatic AA′BB′ spin systems, two aromatic ABX spin systems, and also an aliphatic AB system at *δ*_H_ 6.01 and 4.67 (d, *J* = 11.0 Hz, H-a_2_/b_2_).

**Figure 2:**
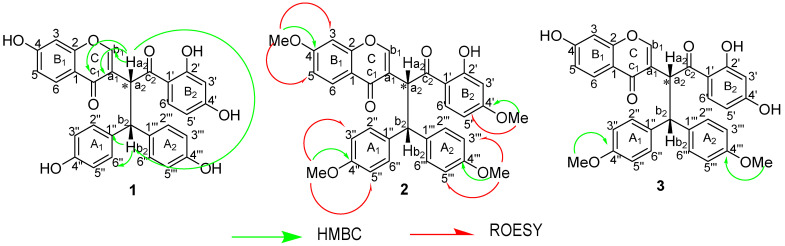
Key HMBC and ROESY correlations of (**1**–**3**).

The ^13^C-NMR spectrum (Table 1) of **1** exhibited resonances of 30 carbon atoms, which were resolved by APT (J-mod) and HSQC experiments, including 15 unusual isoflavone skeleton carbons. Nine of these carbons resonated at *δ*_C_ 177.2; 164.7; 159.5; 157.5; 128.2; 122.7; 116.6; 108.6 and 103.3 and six carbons assigned to the 1,4-disubstituted ring A_1_ at 156.8, 134.9; 130.5 and 116.2. The remaining 15 carbons resonated at *δ*_C_ 204.9, 167.0, 166.6, 156.7, 136.0, 134.4, 129.9, 116.1, 114.4, 109.3, 54.4 and 44.8, suggesting the presence of a dihydrochalcone backbone, indicating that compound **1** is a biflavonoid composed of one unusual isoflavone together with a dihydrochalcone monomer. The connection between Ca_1_ and Ca_2_ and also between C-1″ and C_b2_ was unambiguously assigned by the HMBC correlations (Figure 2) from H-a_2_ (*δ*_H_ 6.01) to C-a_1_ (*δ*_C_ 122.7); C-c_1_ (*δ*_C_ 177.2) and C-b_1_ (*δ*_C_ 157.5) and H-b_2_ (*δ*_H_ 4.67) to C-1″ (*δ*_C_ 134.9) and C-2″ (*δ*_C_ 130.5). The presence of five oxygenated aromatic carbons displayed in the ^13^C-NMR spectrum confirmed the existence of five hydroxyl groups located on benzene rings. These findings supported a Lophirone A skeleton for compound **1** [12,17,25,26,29].

The CD spectral data of compound **1** (Figure S21) exhibited the cotton effects of the electronic transition n→π* and π→π* transitions of the two flavonoid moieties. Successive high-amplitude positive Cotton effects for the n→π* electronic transition at 309 nm and high-amplitude positive Cotton effect for the π→π* transition at 248 nm, respectively, suggest **(*R*)** absolute configuration of the stereogenic center (Ca_2_). The spectrum also displayed a low-amplitude positive Cotton effect for the π→π* at 208 nm, in agreement with the assigned absolute configuration [16]. Moreover, a high-amplitude positive Cotton effect is consistent with the *β*-orientation of proton H-a_2_. Based on the above comprehensive spectroscopic data analysis of the compound, the planar structure of compound **1** was established as depicted in Figure 1 as (*R*)-3-(1-(2′,4′-dihydroxyphenyl)-3,3-bis(4″,4‴-hydroxyphenyl)-1-oxopropan-2-yl)-4-hydroxy-chromenone and named (*R*)-rhizomatobiflavonoid A (Figure 1).

Compound **2** was isolated as a yellow powder eluted with CH_2_Cl_2_/MeOH (20:1) and it gave a positive reaction with Neu’s reagent. Its HR-ESIMS spectral data exhibited a protonated molecular ion peak at *m/z* 567.1274 [M + H]^+^, which corresponds to the molecular mass of C_34_H_30_O_8_ (calc. 567. 3501 [M + H]^+^). The UV and FTIR spectral data for characteristic functional groups of compound **2** were similar to those obtained for compound **1**. When compared to the ^1^H and ^13^C NMR spectral data of compound **1** (Table 1), compound **2** exhibited a unique difference as four of the five hydroxy groups were substituted by methoxy groups, which resonated at *δ*_H_ 3.63, 3.75, 3.78 and 3.63 with the corresponding carbon signals at *δ*c 55.7, 55.6, 56.2 and 54.9. This was further confirmed by the molecular formula of C_34_H_30_O_8_, bearing only one hydroxy group and four methoxy groups compared to compound **1.**

The HMBC cross peaks of the methoxy protons at *δ*_H_ 3.78, 3.75 and 3.63 correlated to the carbon atoms at *δ*_C_ 167.0 (C-4′), 164.9 (C-4), 159.6 (C-4‴) and 159.5 (C-4″), respectively. Further confirmation of the position of the methoxy groups of **2** was supported by the ROESY spectral data, displaying the following main cross-peaks: MeO-4‴/H-3‴, 5‴; MeO-4″/H-3″, 5″; MeO-4ʹ/H-5ʹ and MeO-4/H-5.

As for compound **1,** the CD spectral data of **2** (Figure S22) exhibited a successive high-amplitude positive Cotton effect for the n→π* electronic transition at 305 nm and high-amplitude positive Cotton effect for the π→π* transition at 247 nm, respectively, suggesting the **(*R*)** absolute configuration of the stereogenic center (Ca_2_). The CD spectrum also displayed a low-amplitude positive Cotton effect for the π→π* transition at 207 nm, in agreement with the assigned absolute configuration [16]. Moreover, a high-amplitude positive Cotton effect is consistent with the *β*-orientation of proton H-a_2_. Thus, the structure of **2** was proposed as a (*R*)-3-(1-(2′-hydroxy-4′-methoxyphenyl)-3,3-bis(4″,4‴-dimethoxyphenyl)-1-oxopropan-2-yl)-4-methoxychromenone biflavonoid connected through rare Ca_1_-Ca_2_ and C-1″-Cb_2_ bonds and named (*R*)-rhizomatobiflavonoid B (Figure 1).

Compound **3** was isolated as a yellowish amorphous powder, eluted with CH_2_Cl_2_/MeOH (20:1), and showed UV and FTIR spectral data similar to those of compounds **1** and **2**. The 1D and 2D NMR spectral data revealed a high degree of similarity with compound **1** in chemical shifts as well as the coupling patterns and substituent positions except for both the carbon and proton signals at C-4″ and C-4‴. When compared with the NMR spectral data of compound **1**, compound **3** displayed one proton signal at *δ*_H_ 3.65 (6H, s) in the ^1^H NMR (Table 1) with the respective carbon signals at *δ*_C_ 55.4 and 55.5 in the ^13^C NMR, suggesting the occurrence of two methoxy groups in the chemical structure of compound **3**. The molecular formula of compound **3** was established as C_32_H_26_O_8_ by the ESIMS ion peak at *m/z* 537.3 [M-H]^−^, (calc. 537.1 [M-H]^−^), and also indicated the presence of two additional methoxy groups. However, the position of these two methoxy groups was endorsed by the correlations found in the HMBC spectral data of the first methoxy proton at *δ*_H_ 3.65 with the carbon atom at *δ*_C_ 159.6 (C-4‴) and the second at *δ*_H_ 3.65 with the carbon atom at *δ*_C_ 159.7 (C-4″), which proved that these two methoxy groups were positioned at C-4‴ and C-4″, respectively (Figure 2).

The absolute configuration of compound **3** was identical to **1** and **2** as evidenced by the CD spectrum. From the foregoing observations, compound **3** was characterized as (*R*)-3-(1-(2′,4′-dihydroxyphenyl)-3,3-bis(4″-hydroxy-4‴-methoxyphenyl)-1-oxopropan-2-yl)-4 methoxychromenone and named (*R*)-rhizomatobiflavonoid C (Figure 1).

### 3.2. Biological Activities of Isolated Biflavonoids

The anti-HIV-1 replication and antiplasmodial activities of the biflavonoids bearing hydroxy and methoxy groups were studied. Compound **7** (4′,7-dihydroxy-3′,5-dimethoxyisoflavone), is not a biflavonoid, but was included in the bioactivity studies since it represents the single isoflavone present in the rootbark and can be used as a reference and as a starting point to analyze structure–activity relationships of the compounds that were isolated.

#### 3.2.1. Anti-HIV-1 Integrase Activity

**Figure 3:**
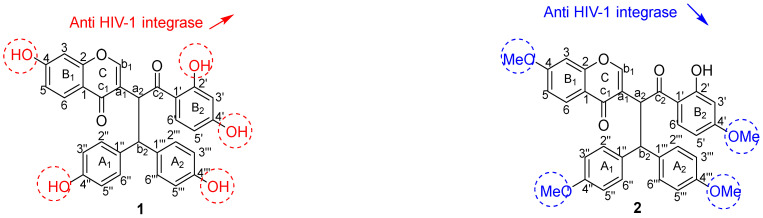
Structure–activity relationships established for anti-HIV-1 effect against the inhibition of the protein integrase of compounds (**1**) and (**2**). Red colors express hydroxyl groups and blue colors express methoxyl groups.

As part of the protease and reverse transcriptase (RT), integrase is the only enzyme that is encoded by HIV-1. This enzyme first cleaves the last two nucleotides from each 3′-end of the linear viral DNA. Chicoric acid, a well-known active inhibitor of HIV-1 integrase, was used as a reference compound during the assay. The results for anti-HIV-1 replication inhibitory activity showed that compound **1** (IC_50_ = 0.047 µM) is the only compound that presents prominent inhibition of the integrase enzyme. This activity is less pronounced than the inhibition obtained for the control compound chicoric acid (IC_50_ = 0.006 µM), a pure competitive inhibitor of HIV-1 integrase used clinically in the early stages of viral replication. The results suggest that a higher number of free hydroxy groups seems to increase the inhibitory activity of HIV-1 integrase of the compound, since compound **1,** which is a biflavonoid, is more active than its respective methoxy derivatives (compounds **2, 3, 4** and **5**) (Table 2). It can, therefore, be suggested from the structure–activity relationships that unusual biflavonoids consisting of an isoflavone unit fused with a dihydrochalcone skeleton with a higher number of free hydroxy groups increase the inhibition of HIV-1 replication, while the presence of methoxy groups as substituents decreases the inhibition (Figure 3). However, this affirmation has been further supported by molecular docking data obtained in this study.

#### 3.2.2. Antiplasmodial Activity

**Figure 4:**
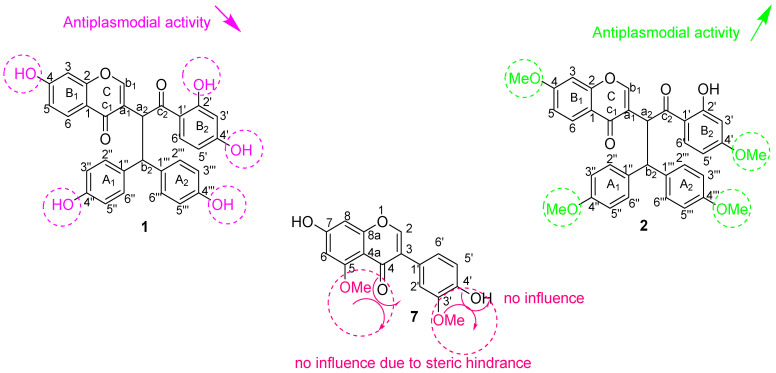
Structure–activity relationships established for antiplasmodial effect against chloroquine-sensitive strain of the malaria parasite *Plasmodium falciparum* NF*54* of compounds (**1**), (**2**) and (**7**).

The antiplasmodial activity of the studied compounds did show promising IC_50_ values for compounds **2** (IC_50_ = 4.60 µM) and **5** (IC_50_ = 5.11 µM), and less activity for compounds **3** (IC_50_ = 7.86 µM) and **4** (IC_50_ = 8.20 µM). The activity was, however, not comparable to that of the reference compounds (artesunate and chloroquine (CQ)) (Table 3). It was also observed that compounds **1, 6** and **7** displayed no activity against the chloroquine-sensitive strain of the malaria parasite (*P. falciparum* NF*54*)**.** These results suggested some interesting structure–activity relationships (Figure 4). Compound **2**, with the highest number of methoxy groups, also exhibited the highest activity, suggesting that methoxy groups might play an important role in the molecule’s mode of action against the chloroquine-sensitive strain of the malaria parasite *(Plasmodium falciparum* NF*54*)**,** while compound **1**, which contains no methoxy groups, did not present any activity against *P. falciparum* NF*54* (Table 3). The influence of a methoxy group in biflavonoid compounds’ antiplasmodial activity was not a surprise because a similar trend was observed in our previous report [12]. The presence of methoxy groups with steric hindrances in the molecule does seem to reduce the compound’s antiplasmodial activity. This supports the lack of antiplasmodial activity of compound **7** (isoflavonoid). This conclusion has been confirmed by molecular docking.

**Figure 5:**
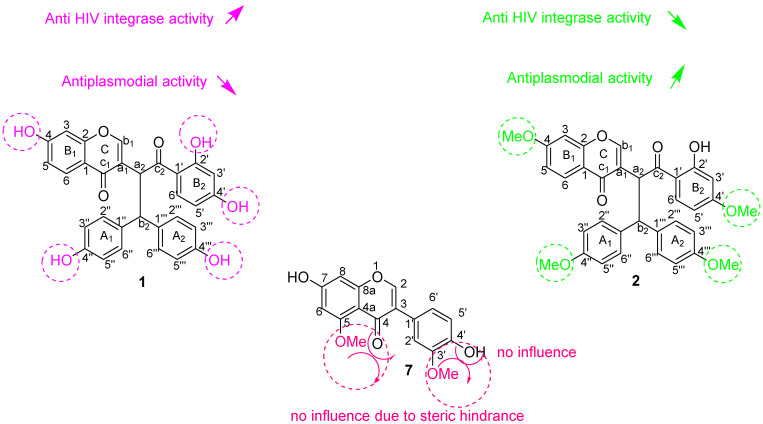
Summary of the most relevant structure–activity relationships established by compounds (**1**), (**2**) and (**7**).

From the anti-HIV-1 integrase and antiplasmodial activities exhibited by these isolated compounds, some important structure–activity relationships can be established for all the tested compounds, and the most important are highlighted and summarized in Figure 5.

As shown in Figure 5, the presence and number of free hydroxy groups seem responsible for the inhibitory activity of HIV-1 integrase, while methoxy groups seem to have the opposite effect. Sterically hindered methoxy groups, as illustrated in Figure 5 for compound **7**, leads to the absence of activity against *Plasmodium falciparum* strain NF*54*.

#### 3.2.3. Molecular Docking

**Figure 6:**
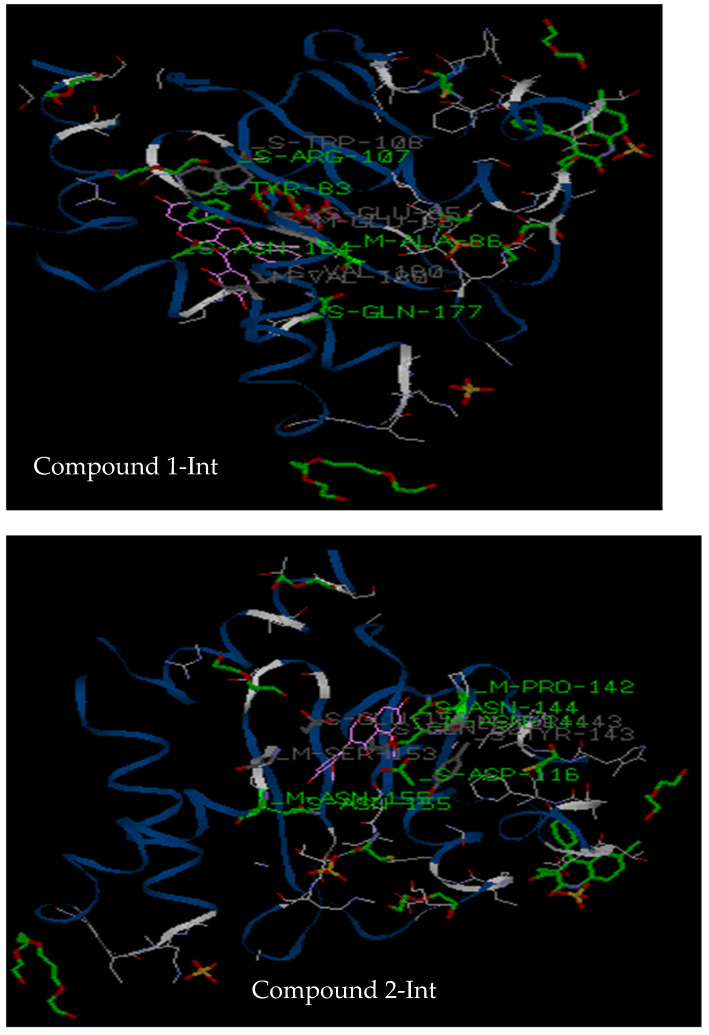

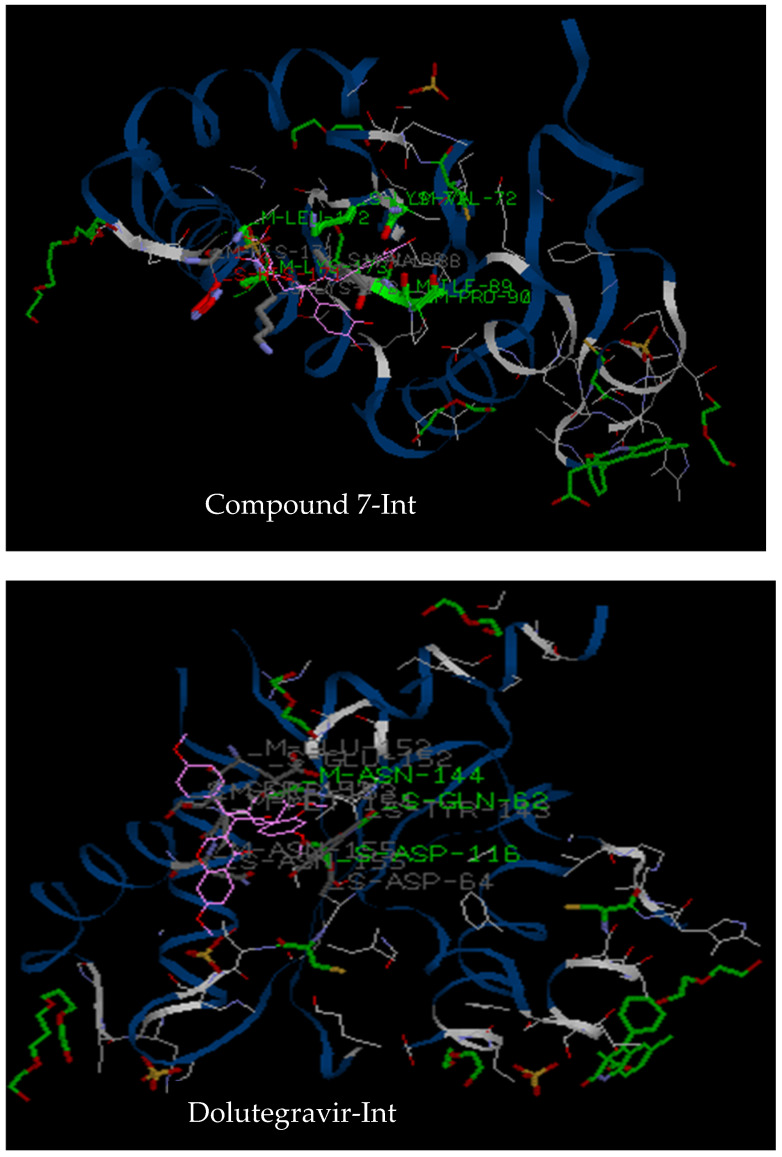

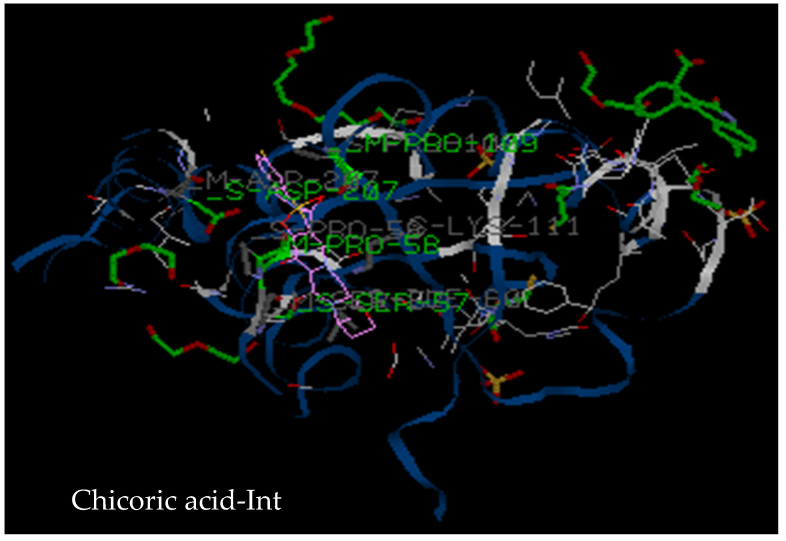
Interaction of compounds **1**, **2**, **7**, Dolutegravir and chicoric acid with integrase protein (PDB ID: 3LPT). Compound **1**: (*R*) rhizomatobiflavonoid A; Compound **2**: (*R*) rhizomatobiflavonoid B; Compound **7**: Gerontoisoflavone A. Int: integrase receptor.

**Figure 7:**
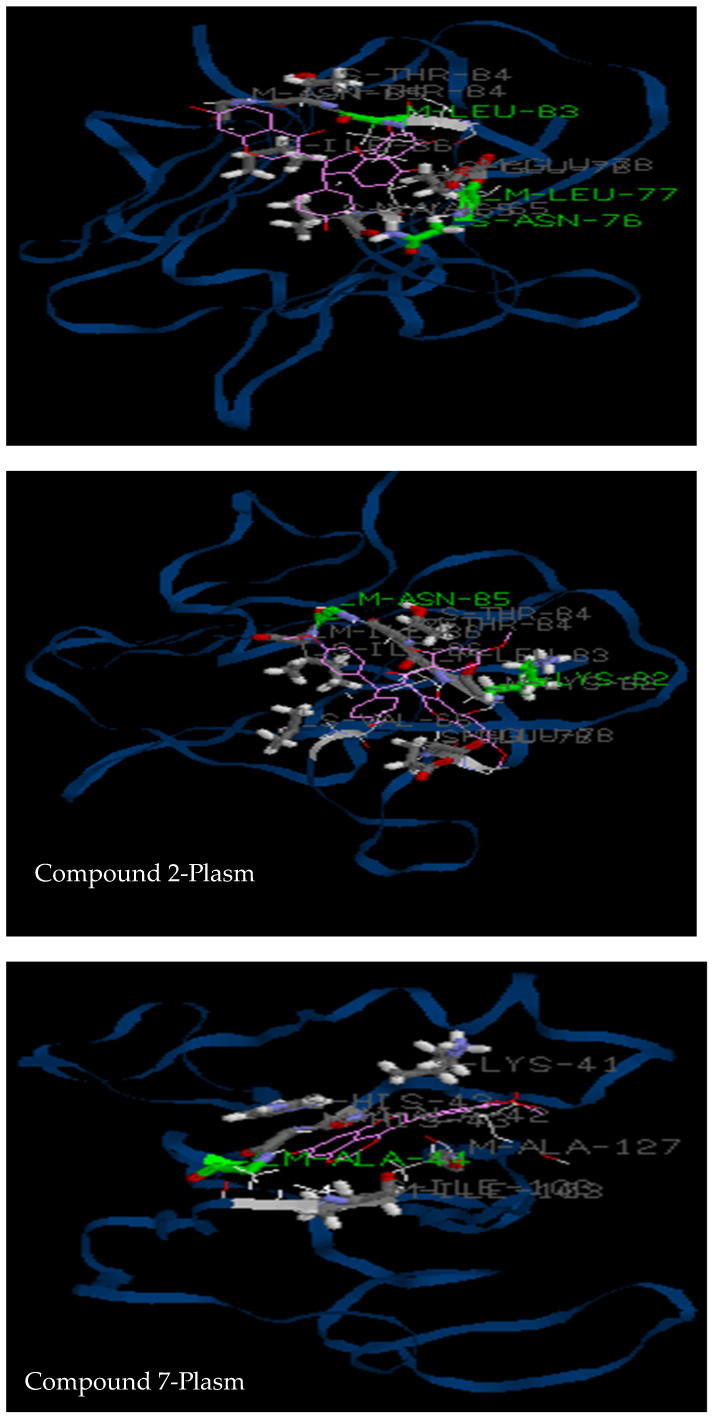

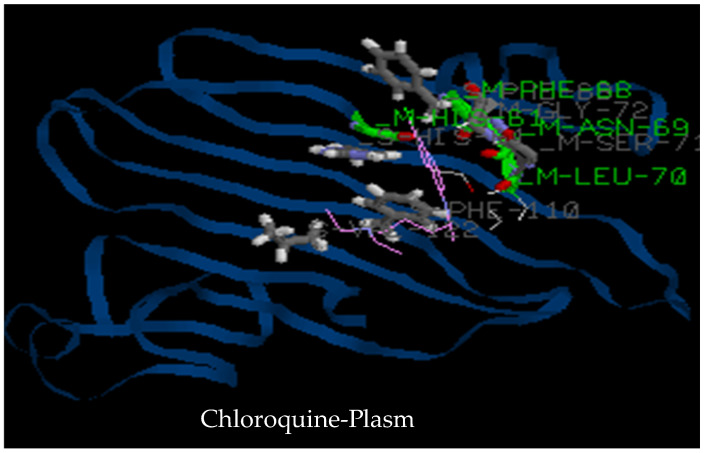
Interaction of compounds **1, 2, 7** and chloroquine with active cavities of *Plasmodium falciparum* protein receptor, PDB ID: 2LOE. Compound **1**: (*R*) rhizomatobiflavonoid A; Compound **2**: (*R*) rhizomatobiflavonoid B; Compound **7**: Gerontoisoflavone A. Plasm: *Plasmodium* receptor.

In the next experiments, in silico analysis with the isolates was conducted using the Molegro software. The best poses were selected from each docking analysis and pictures saved, as shown in Figure 6 and Figure 7. Moreover, different interactions of the ligands with amino acids, including hydrogen bonds, van der walls interactions and total binding energies, were calculated and represented in Table 4 and Table 5 below. The software has also identified all the amino acids involved in the binding and their respective positions, as shown in Table 6 and Table 7, respectively, for integrase and *Plasmodium* receptors.

The aim of docking analysis was to predict the binding capacities of isolated compounds on integrase and *Plasmodium* receptors; as well as the amino acids involved in the binding pockets. Therefore, compounds **1** to **7** were docked against integrase and the *Plasmodium*-6 cysteine s48/45 domain, two membrane proteins necessary for viral and parasite invasion into human cells. As far as integrase enzyme is concerned, compounds **3**, **4**, **5** and **6** showed very low binding scores with the enzyme (data not shown); suggesting no interaction of these compounds with the binding site of the enzyme. This result corroborates the observations obtained in vitro with no inhibition detected with these compounds. However, both compounds **1** and **2** were found bound to the active cavity of integrase receptor protein and showed binding with amino acids of the enzyme’s active site. The binding energy of compound 1 with amino acids (Tyr83, Ala86, Arg107, Asn184, Tyr83, Glu85, Trp108, Glu177, Val180, Asn184) was compared with the binding energy of Dolutegravir with amino acids (Asp116, Asn144, Gln62, Glu152, Ser153, Met154, Asn155Arg657, Asp655) and of chicoric acid with amino acids (Asp116, Asn144, Gln62) (Table 5). The results showed that compounds **1** and **2** bind to integrase with the highest score and strongest energy (MolDock score: −121.35 and −131 Kcal/mol, respectively) in comparison with chicoric acid and Dolutegravir (MolDock score: −116 and −100 Kcal/mol respectively). As observed in vitro, compound **1** seems to be a promising inhibitor of integrase with an IC_50_ close to that of chicoric acid. Binding poses showed that compound **1** establishes five hydrogen bonds with the active site’s amino acids while chicoric acid has six and Dolutegravir only three, suggesting that in silico and in vitro observations are quite similar. Contrary to what was observed in vitro, compound **2** exhibited an interesting binding score in docking, suggesting that other factors may influence its activity in vitro but deserves further investigations to confirm its antiviral activity. Docking of compound **7** also showed a lower binding score in comparison to compounds **1**, **2** and the reference compounds, confirming the result obtained in vitro (Table 2).

As described above, docking analyses were also performed with the *Plasmodium*-6-cysteine s48/45 domain, which is a membrane protein responsible for parasite entry into human cells. An analysis of binding interactions revealed that compounds **1** and **2** displayed high affinity for *Plasmodium* protein PDB ID: 2LOE with three and two hydrogen bonds with seven amino acids, in comparison to one hydrogen bond with chloroquine. The same observation was obtained for the binding score with −125 Kcal/mol for compounds **1** and **2** and −84 Kcal/mol for chloroquine. As it was observed in vitro, compound 2 seems to be a promising antiplasmodial compound. Compounds **3** and **4** showed no binding with the enzyme (data not shown). Contrary to in vitro results, compound **1** showed a high binding score with the enzyme in comparison to the reference drug chloroquine, suggesting that further in vitro and in vivo experiments should be performed in the future to confirm its antiplasmodial effect. Several plant compounds isolated from *Dioscorea bulbifera* showed antiplasmodial effects in the docking analysis, thereby supporting this strategy of in silico analysis as a platform of discovering new lead compounds against malaria [30].

These results confirm the potential inhibitory effect of compounds **1** and **2** for HIV and malaria treatment.

## 4. Conclusions and Recommendations

The phytochemical investigation of the root barks of *Ochna rhizomatosa* led to the isolation and structure elucidation of six biflavonoids (**1**–**6**) and one isoflavonoid (**7**). The anti-HIV-1 replication and antiplasmodial activities were assessed during this study. Compound **1** exhibited a noteworthy inhibition of HIV-1 integrase (IC_50_ = 0.047 µM), whereas compound **2** displayed the highest antiplasmodial activity (IC_50_ = 4.60 µM)**.** Simultaneously, a structure–activity relationship was established. The presence and number of free hydroxy groups seem responsible for the inhibitory activity of HIV-1 integrase, while methoxy groups seem to have the opposite effect. Sterically hindered methoxy groups lead to the absence of activity against *Plasmodium falciparum* strain NF*54*. Docking studies showed that compounds **1** and **2** are good inhibitors for both integrase and *Plasmodium* 6-cysteine s48/45 domain receptor proteins with a binding score higher than control drugs and well-known inhibitors, indicating their potential effect as lead compounds against HIV and malaria. These results indicate the necessity to confirm our observations in vivo, which will be the direction of our future research.

## Figures and Tables

**Table 1:** ^1^H NMR (400 MHz) and ^13^C NMR (100 MHz) data of 1, 2 and 3 (MeOH-*d*_4_).

		1			2			3
No.	*δ*_C_	*δ*_H_ (*J* in Hz)	No.	*δ*_C_	*δ*_H_ (*J* in Hz)	No.	*δ*_C_	*δ*_H_ (*J* in Hz)
B_1_-1	108.6	-	B_1_-1	108.6	-	B_1_-1	108.6	-
2	159.5	-	2	159.5	-	2	159.5	-
3	103.3	6.71 (d, 2.5)	3	103.3	6.71 (d, 2.5)	3	103.3	6.71 (d, 2.5)
4	164.7	-	4	164.9	-	4	164.9	-
5	116.6	6.85 (dd, 2.5, 9.0)	5	116.6	6.85 (dd, 2.5, 9.0)	5	116.6	6.85 (dd, 2.5, 9.0)
6	128.3	7.88 (d, 9.0)	6	128.3	7.88 (d, 9.0)	6	128.3	7.88 (d, 9.0)
C-_1_	177.2	-	C-_1_	177.2	-	C-_1_	177.2	-
a_1_	122.7	-	a_1_	122.7	-	a_1_	122.7	-
b_1_	157.5	8.23 (s)	b_1_	157.5	8.23 (s)	b_1_	157.5	8.23 (s)
B_2_-1′	114.4	-	B_2_-1′	114.4	-	B_2_-1′	114.4	-
2′	166.6	-	2′	166.6	-	2′	166.6	-
3′	103.6	6.14 (d, 2.0)	3′	103.6	6.14 (d, 2.0)	3′	103.6	6.14 (d, 2.0)
4′	167.0	-	4′	167.0	-	4′	167.0	-
5′	109.3	6.34 (dd, 2.0, 9.0)	5′	109.3	6.34 (dd, 2.0, 9.0)	5′	109.3	6.34 (dd, 2.0, 9.0)
6′	136.0	8.15 (d, 9.0)	6′	136.0	8.15 (d, 9.0)	6′	136.0	8.15 (d, 9.0)
C-_2_	204.9	-	C-_2_	204.9	-	C-_2_	204.9	-
a_2_	44.8	6.01 (d, 11.0)	a_2_	44.8	6.01 (d, 11.0)	a_2_	44.8	6.01 (d, 11.0)
b_2_	54.4	4.67 (d, 12.0)	b_2_	54.4	4.67 (d, 12.0)	b_2_	54.4	4.67 (d, 12.0)
A_1_-1″	134.4	-	A_1_-1″	134.4	-	A_1_-1″	134.4	-
2″	129.9	7.13 (d, 8.5)	2″	129.9	7.13 (d, 8.5)	2″	129.9	7.13 (d, 8.5)
3″	116.1	6.65 (d, 8.5)	3″	116.1	6.65 (d, 8.5)	3″	116.1	6.65 (d, 8.5)
4″	156.7	-	4″	159.5	-	4″	159.7	-
5″	116.1	6.65 (d, 8.5)	5″	116.1	6.65 (d, 8.5)	5″	116.1	6.65 (d, 8.5)
6″	129.9	7.13 (d, 8.5)	6″	129.9	7.13 (d, 8.5)	6″	129.9	7.13 (d, 8.5)
A_2_-1‴	134.9	-	A_2_-1‴	134.9	-	A_2_-1‴	134.9	-
2‴	130.5	7.17 (d, 8.5)	2‴	130.5	7.17 (d, 8.5)	2‴	130.5	7.17 (d, 8.5)
3‴	116.2	6.60 (d, 8.5)	3‴	116.2	6.60 (d, 8.5)	3‴	116.2	6.60 (d, 8.5)
4‴	156.8	-	4‴	159.6	-	4‴	159.6	-
5‴	116.2	6.60 (d, 8.5)	5‴	116.2	6.60 (d, 8.5)	5‴	116.2	6.60 (d, 8.5)
6‴	130.5	7.17 (d, 8.5)	6‴	130.5	7.17 (d, 8.5)	6‴	130.5	7.17 (d, 8.5)
OH	-	-	4‴-OMe	54.9	3.63	4‴-OMe	55.4	3.65
OH	-	-	4″-OMe	55.7	3.63	4-OMe	55.5	3.65
OH	-	-	4′-OMe	56.2	3.78	OH	-	-
OH	-	-	4-OMe	55.6	3.75	OH	-	-
OH		-	OH	-	-	OH	-	-

**Table 2:** Anti-HIV-1 integrase activity of the compounds (**1**–**7**) of *Ochna rhizomatosa.*

Compounds	Assembly IC_50_ (μM)
**1**	0.047 ± 0.021 ^b^
**2**	-
**3**	-
**4**	-
**5**	-
**6**	-
**7**	-
**Chicoric Acid**	0.006 ± 0.002 ^a^

Legend. Values with the same letters are statistically identical, while those with different letters are statistically different with a threshold value of *p* < 0.05.

**Table 3:** Antiplasmodial activity of the compounds (**1**–**7**) of *Ochna rhizomatosa.*

Compounds	NF*54*: IC_50_ (μM)
**1**	-
**2**	4.60 ± 6.09 ^b^
**3**	7.86 ± 5.12 ^b^
**4**	8.20 ± 0.93 ^b^
**5**	5.11 ± 13.7 ^b^
**6**	-
**7**	-
**Chloroquine (CQ)**	0.006 ± 0.002 ^a^
**Artesumate**	0.002 ± ND

Legend. Values with the same letters are statistically identical, while those with different letters are statically different with a threshold value of *p* < 0.05.

**Table 4:** Docking scores of biflavonoids on integrase 3LPT.

Compound/Drug	Energy (Kcal/mol)	VDW (Kcal/mol)	Hbond (Kcal/mol)	Elec (Kcal/mol)
Compound **1**	−121.8	−96.54	−24.46	0
Compound **2**	−131.88	−116.91	−14.97	0
Compound **7**	−92.85	−68.24	−24.6	0
Chicoric acid	−116.06	−83.27	−28.28	−4.52
Dolutegravir	−100.27	−77.05	−23.22	0

**Table 5:** Docking scores of biflavonoids on *Plasmodium.*

Compound/Drug	Energy (Kcal/mol)	VDW (Kcal/mol)	Hbond (Kcal/mol)	Elec (Kcal/mol)
Compound **1**	−125.33	−107.75	−17.59	0
Compound **2**	−124.95	−116.11	−8.84	0
Compound **7**	−90.99	−85.06	−5.93	0
Chloroquine	−84.48	−69.36	−15.11	0

**Table 6:** Receptor–ligand interactions of screened drugs with Integrase.

Target	Compound 1	Compound 2	Compound 7	Chicoric Acid	Dolutegravir
3LPT	Tyr83, Ala86, Arg107, Asn184, Tyr83, Glu85, Trp108, Glu177, Val180, Asn184 (5 hydrogen bonds)	Gln62, Asp116, Asn 144, Asp64, Tyr143, Glu152, Ser153, Met154, Asn155 (3 hydrogen bonds)	Asp116, Pro142, Asn144, Asn155, Glu62, Tyr143, Glu152, Ser153 (4 hydrogen bonds)	His 171, Lys71, Val72, Ile89, Pro90, His171, Lys 173, Leu172 (6 hydrogen bonds)	Asp116, Asn144, Gln62, Glu152, Ser153, Met154, Asn155 (3 hydrogen bonds)

**Table 7:** Receptor–ligand interactions of screened drugs with *Plasmodium.*

Target	Compound 1	Compound 2	Compound 7	Chloroquine
2LOE	Asn76, Leu77, Leu83, Val65, Glu78, Thr84, Asn85, Ile86 (Hydro 3)	Lys82, Asn85, Val65, Glu78, Leu83, Thr84, Ile86 (Hydro2)	Ala44, Lys41, Ala42, His43, Ile103, Ala127 (Hydro 1)	His61, Phe68, Asn69, Leu70, Ser71, Gly72, Phe110, Val122

## Data Availability

Data are contained within the article or Supplementary material.

## Supplementary Materials

The following supporting information can be downloaded at: https://www.mdpi.com/article/10.3390/pharmaceutics14081701/s1, Figure S1–S22: HRESIMS, NMR and CD spectra of new compounds.
