# Supplementary material for: In Vitro and In Silico Potential Inhibitory Effects of New Biflavonoids from Ochna rhizomatosa on HIV-1 Integrase and Plasmodium falciparum
Source: Pharmaceutics. 2022 Aug 15;14(8):1701. doi: 10.3390/pharmaceutics14081701 (PMC9414862; doi:10.3390/pharmaceutics14081701)
Supplement: Supplementary file 1 [file pharmaceutics-14-01701-s001.zip › pharmaceutics-1794789-supplementary.pdf]

pharmaceutics

MDPI

Article

In Vitro and In Silico Potential Inhibitory Effects of New Biflavonoids from *Ochna rhizomatosa* on HIV-1 Integrase and *Plasmodium falciparum*

Angélique Nicolas Messi [1,2,3,*], Susan Lucia Bonnet [2], Brice Ayissi Owona [4], Anke Wilhelm [2], Eutrophe Le Doux Kamto [1], Joseph Thierry Ndongo [5], Xavier Siwe-Noundou [6,*], Madan Poka [6], Patrick H. Demana [6], Rui W. M. Krause [7], Joséphine Ngo Mbing [1], Dieudonné Emmanuel Pegnyemb [1] and Christian G. Bochet [3]

NMR Data of the new isolated biflavonoids

Compound 1

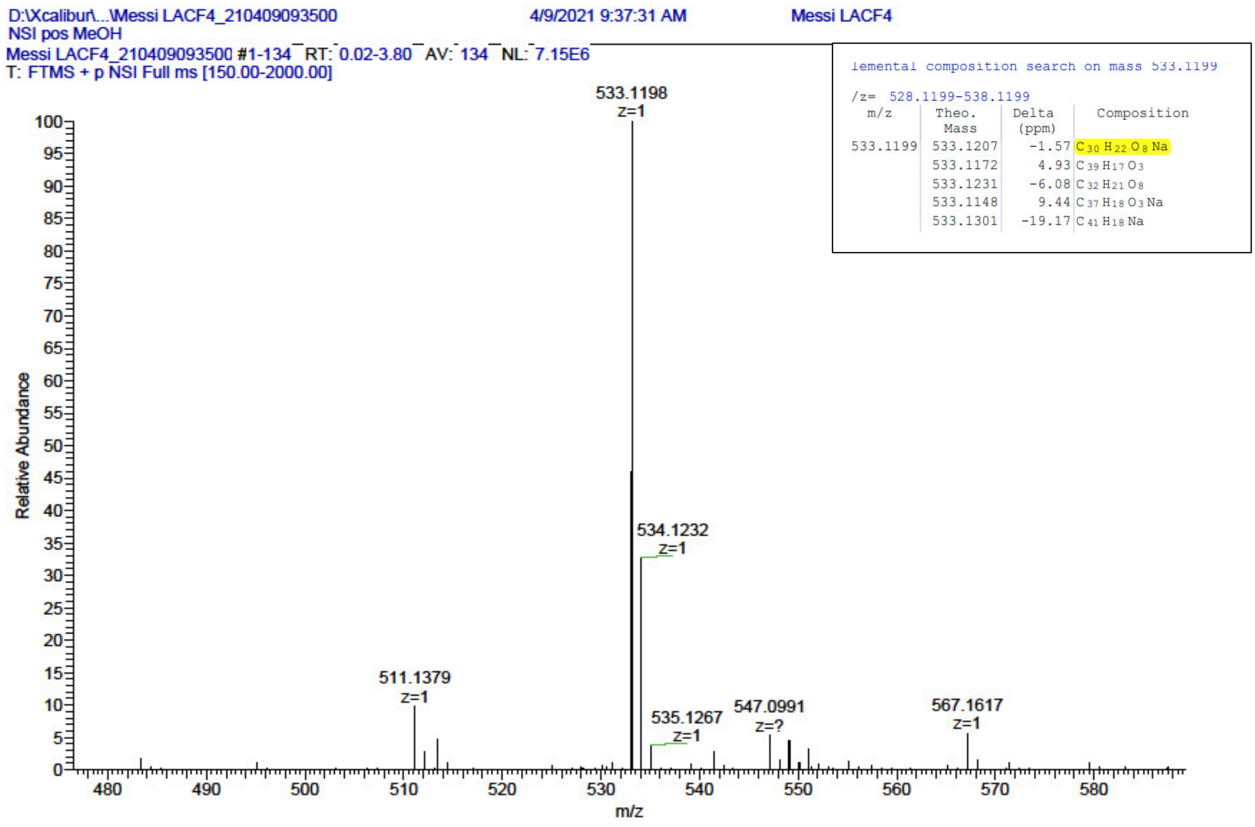

Figure S1. HRESIMS spectrum of compound **1**.

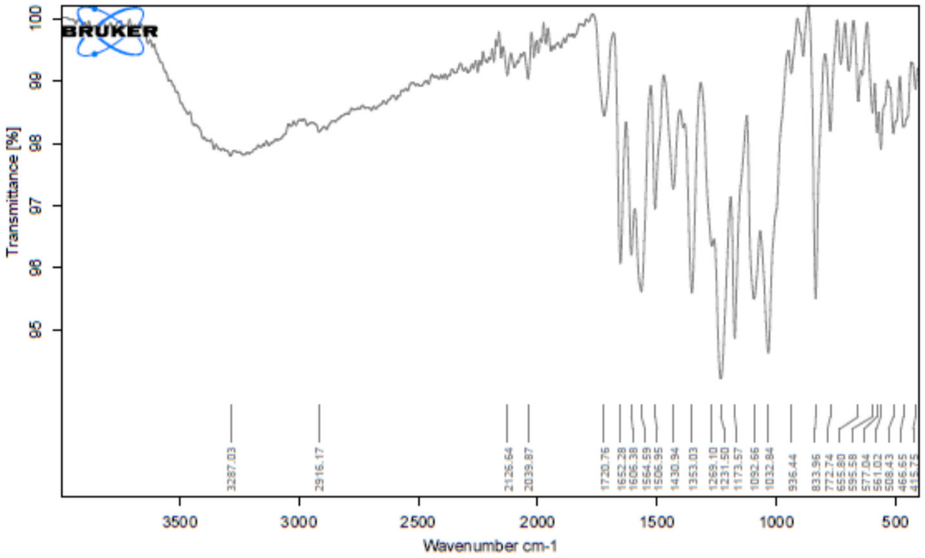

Figure S2. IR spectrum of compound **1**.

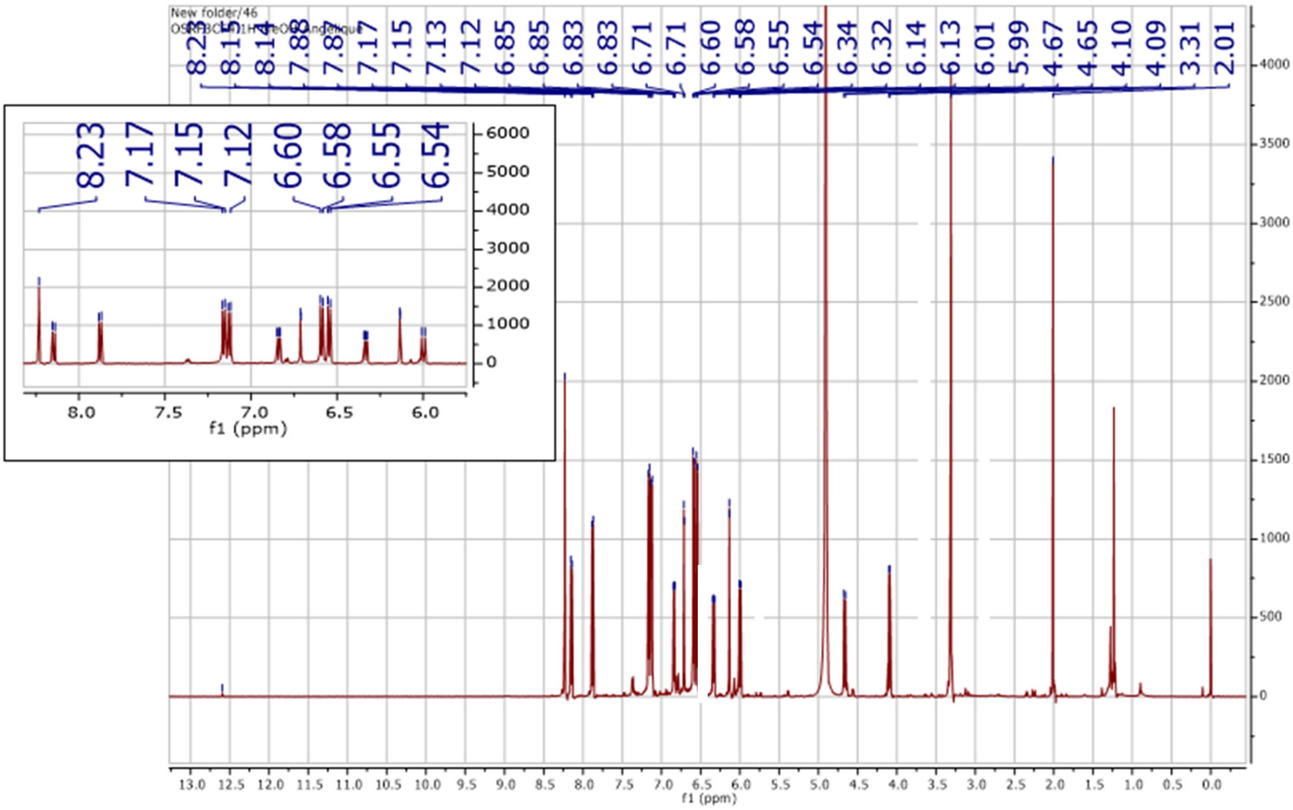

Figure S3: ^{1}H NMR (400 MHz, MeOH-*d*4) spectrum of compound **1.**

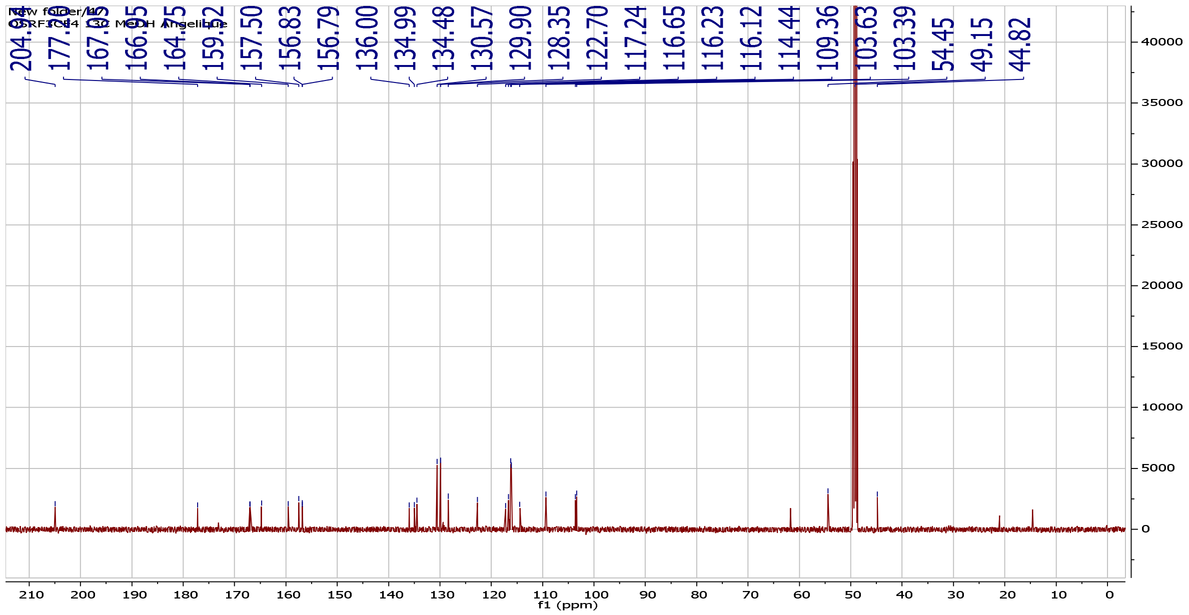

Figure S4. ^{13}C NMR (100 MHz, MeOH-d_4) spectrum of compound **1.**

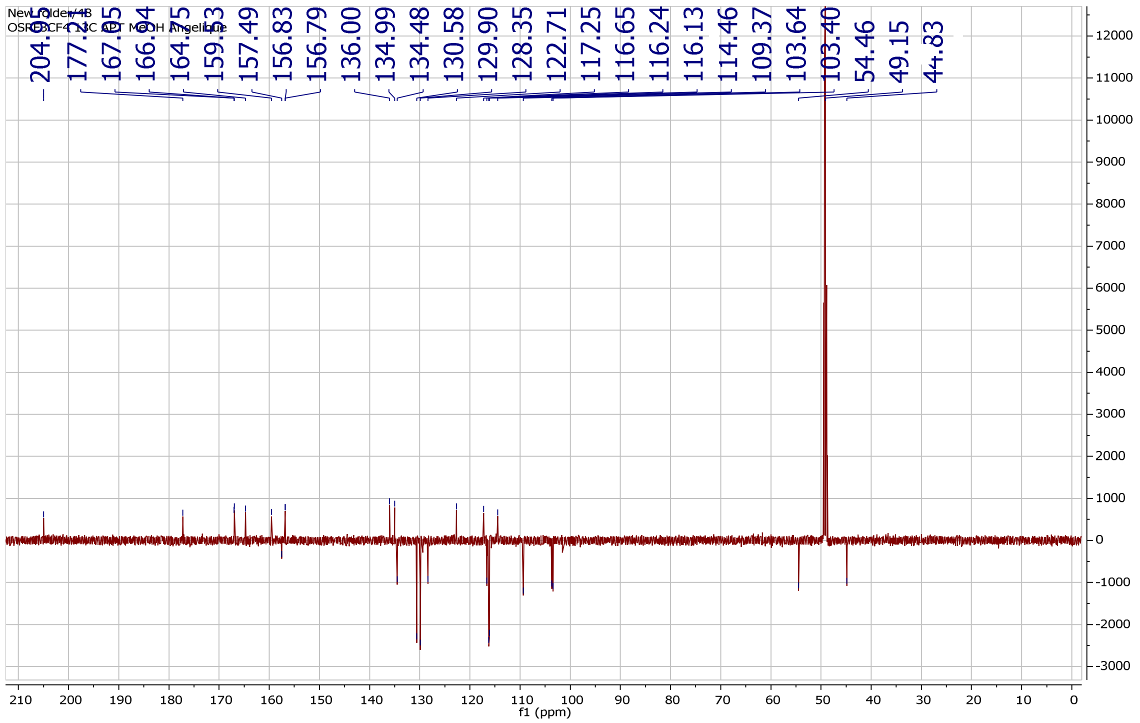

Figure S5. APT (100 MHz, MeOH-d_4) spectrum of compound **1.**

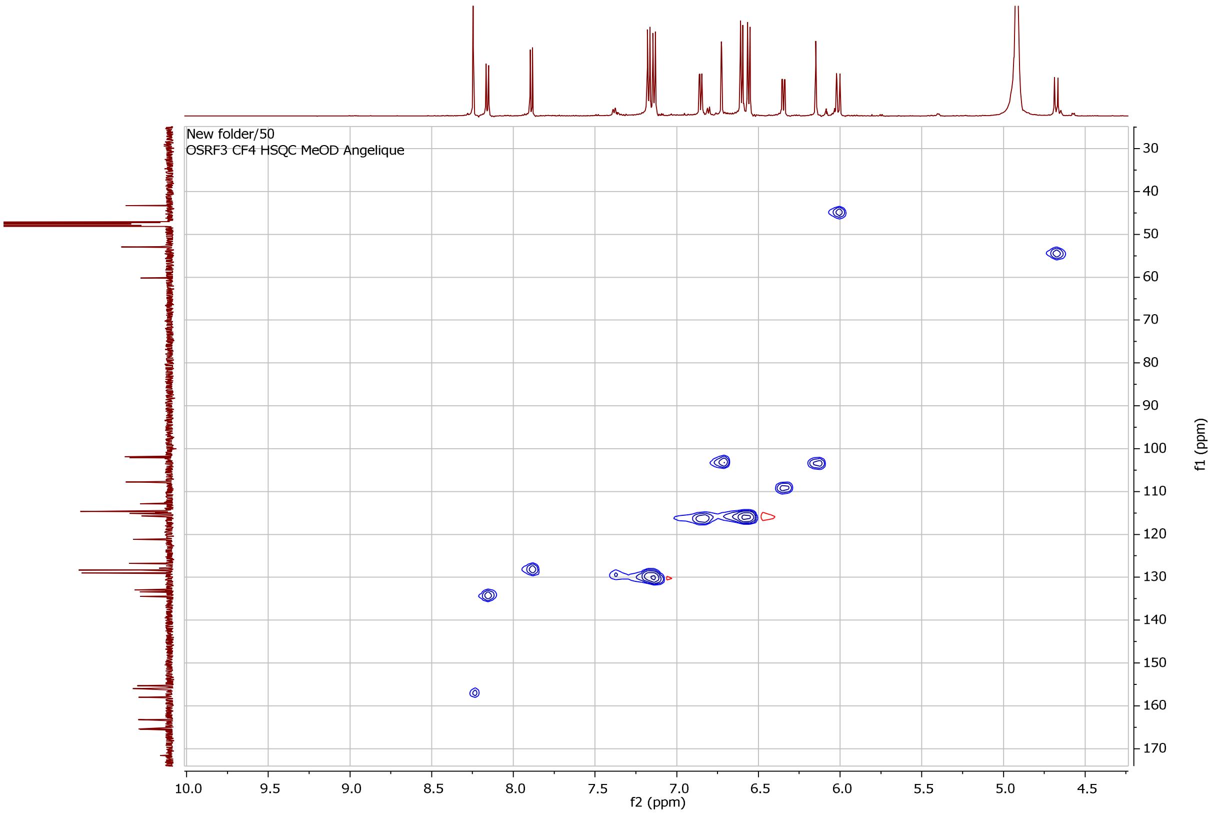

Figure S6. HSQC spectrum of compound **1.**

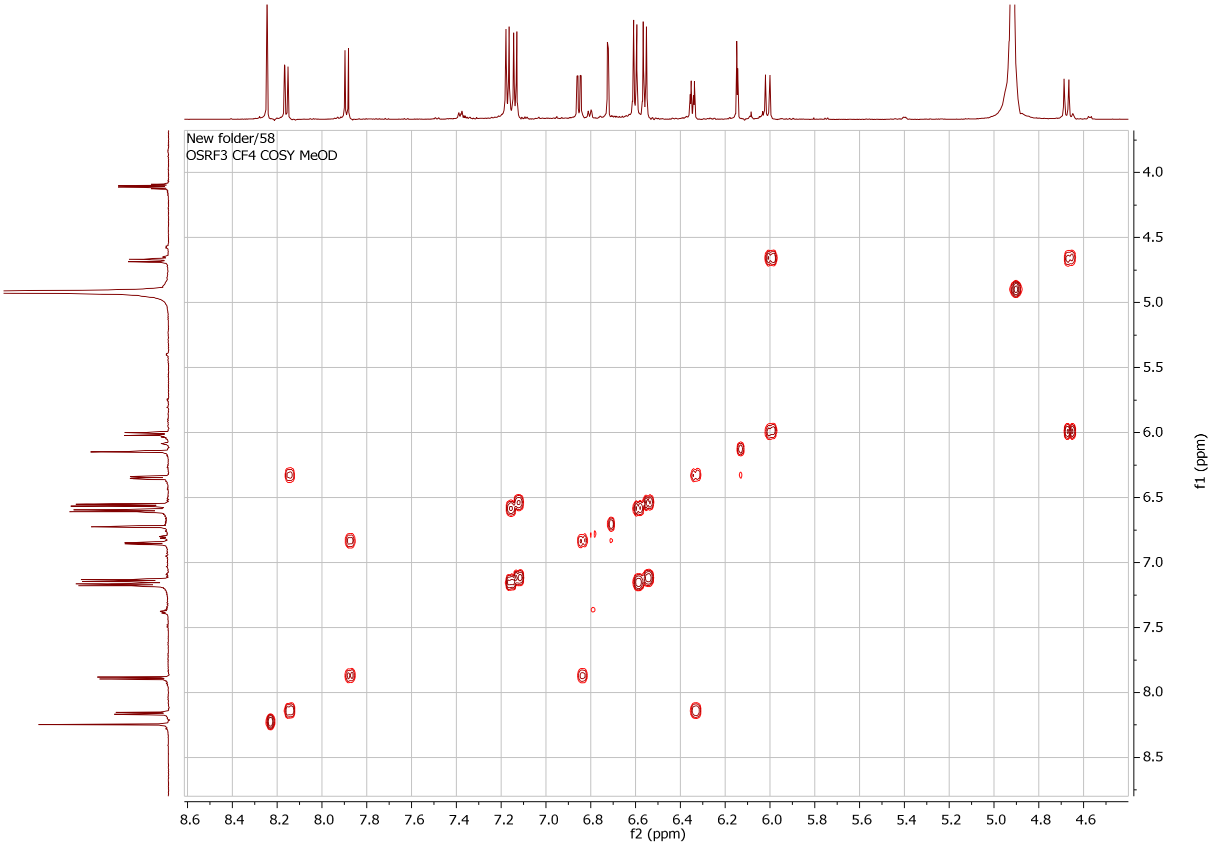

Figure S7. COSY spectrum of compound **1.**

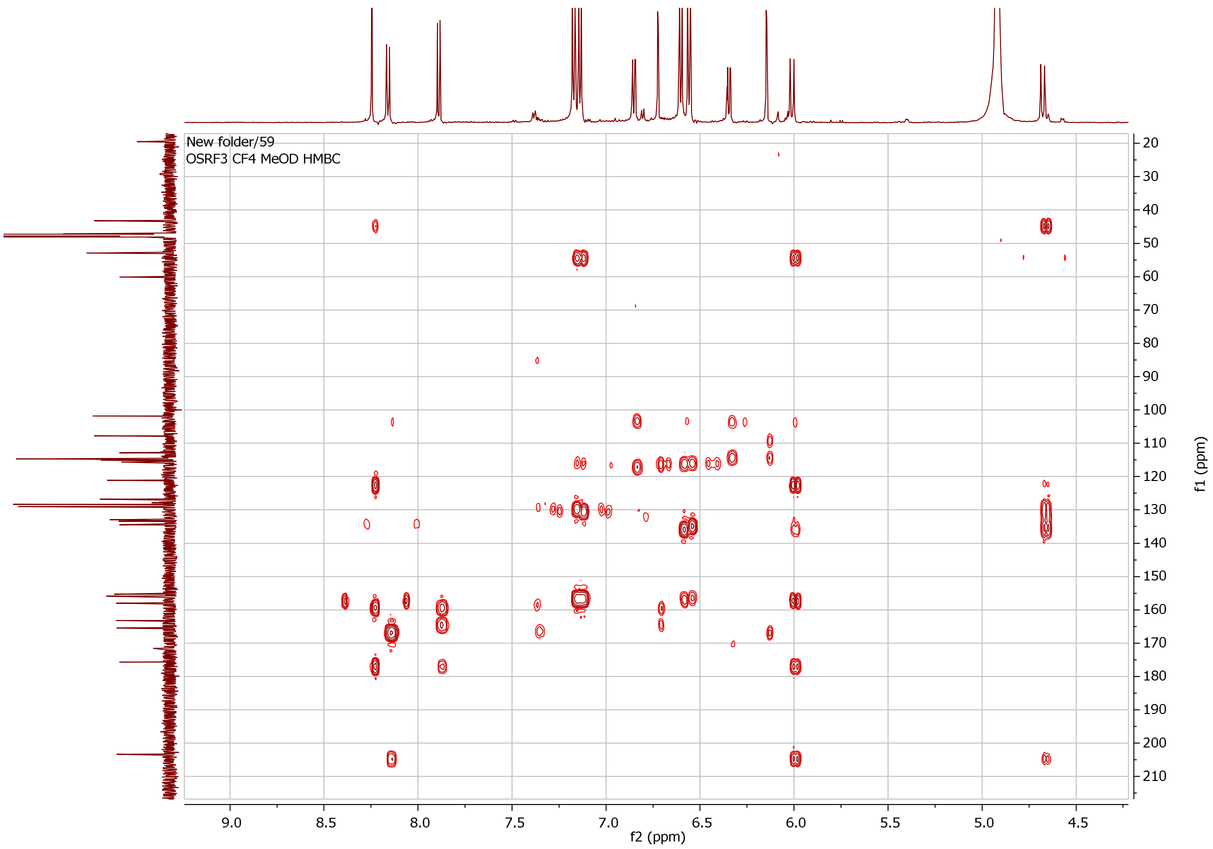

Figure S8. HMBC spectrum of compound **1.**

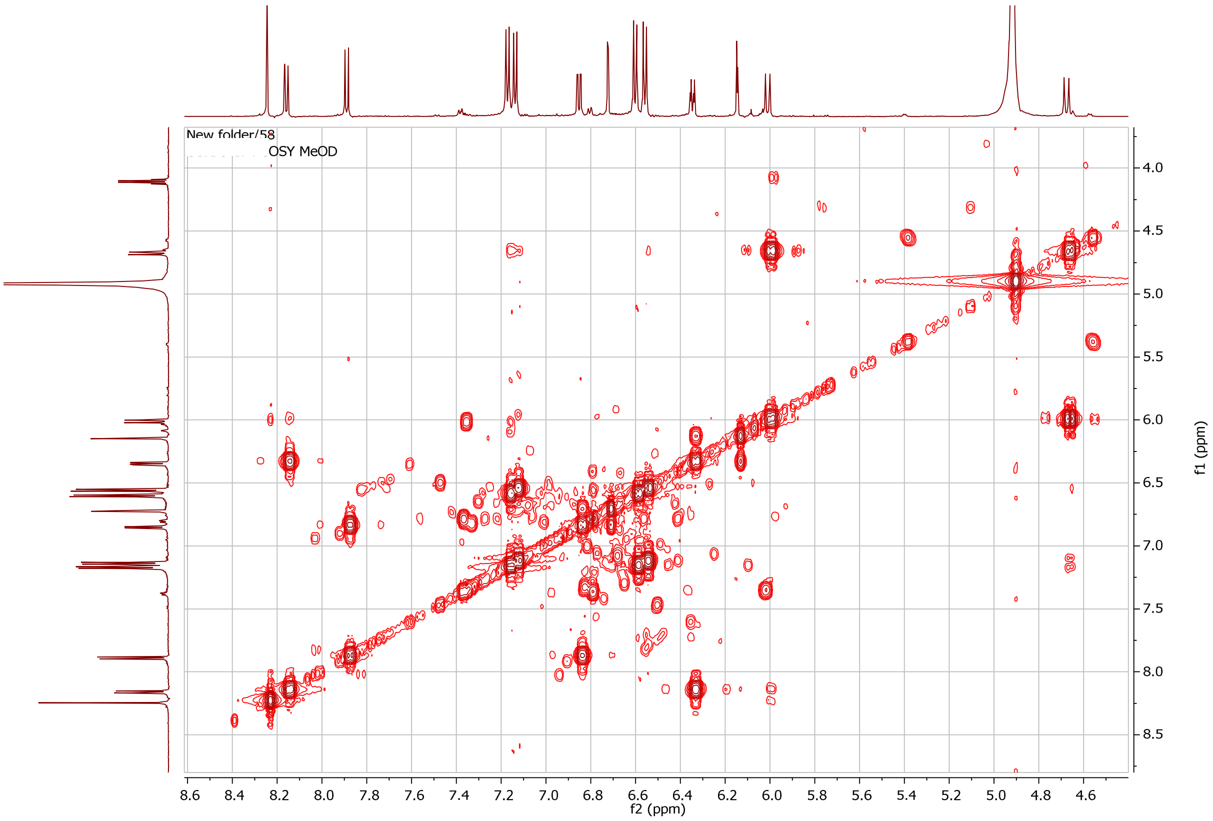

Figure S9. ROESY spectrum of compound **1.**

Compound 2

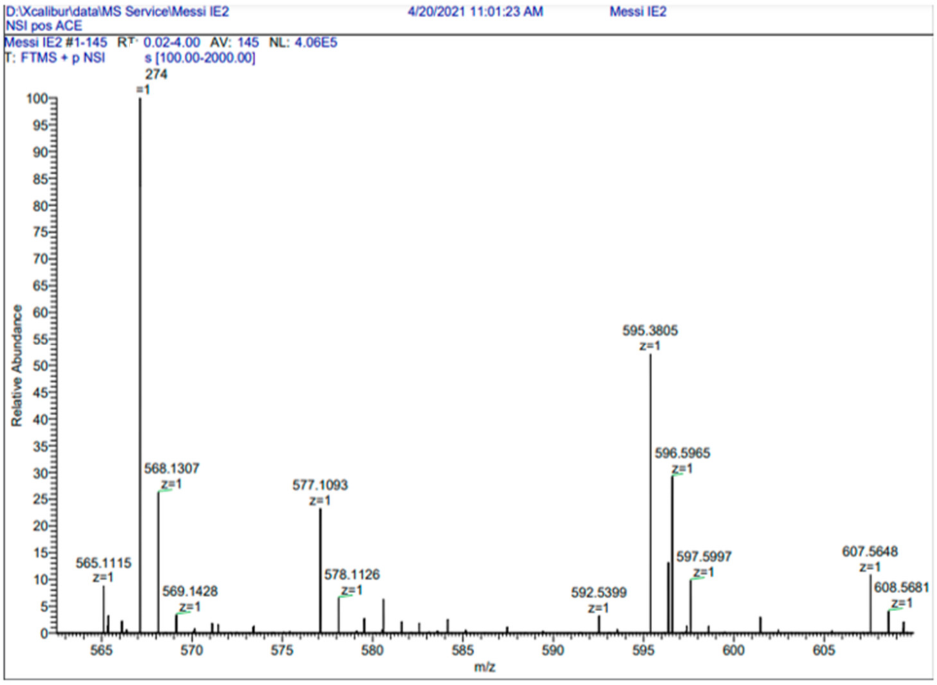

Figure S10. ESIMS Spectrum of compound **2.**

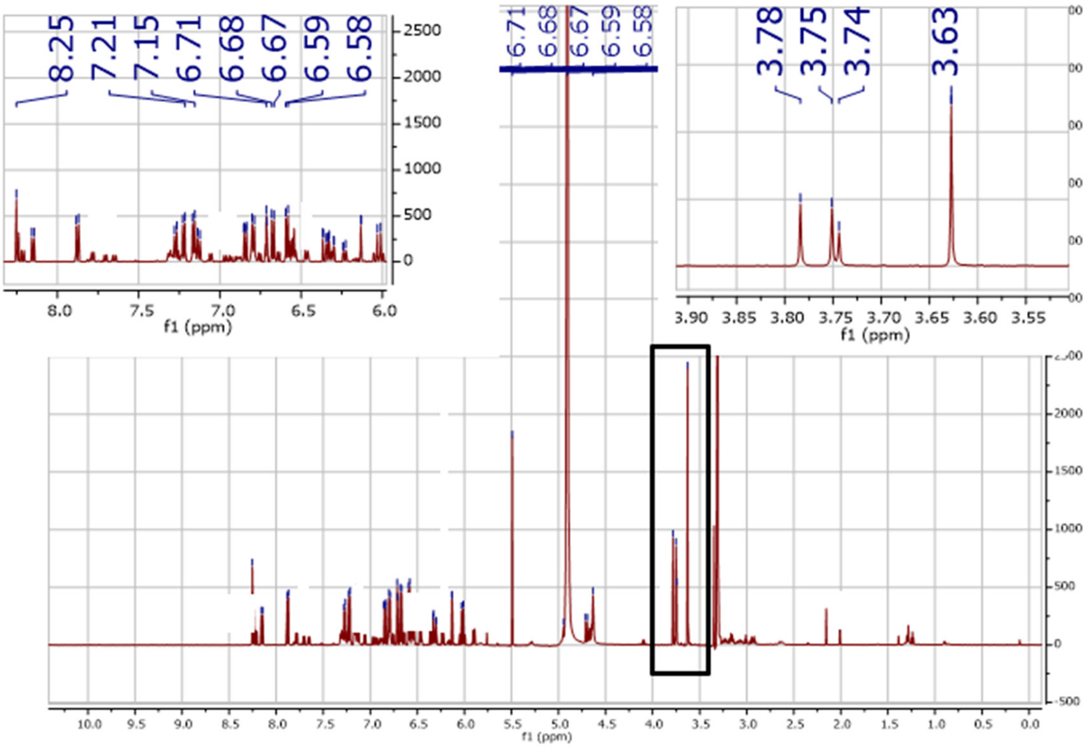

Figure S11. ^{1}H NMR (400 MHz, MeOH-d_4) spectrum of compound **2.**

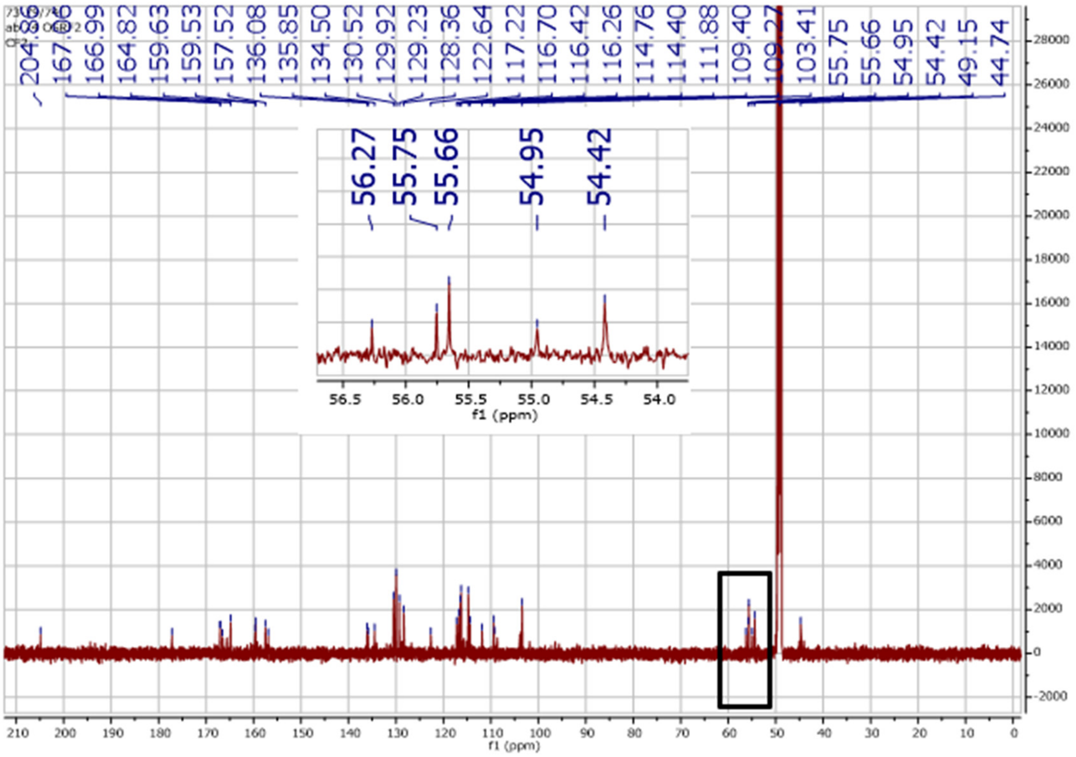

Figure S12. ^{13}C NMR (100 MHz, MeOH-d_4) spectrum of compound **2**.

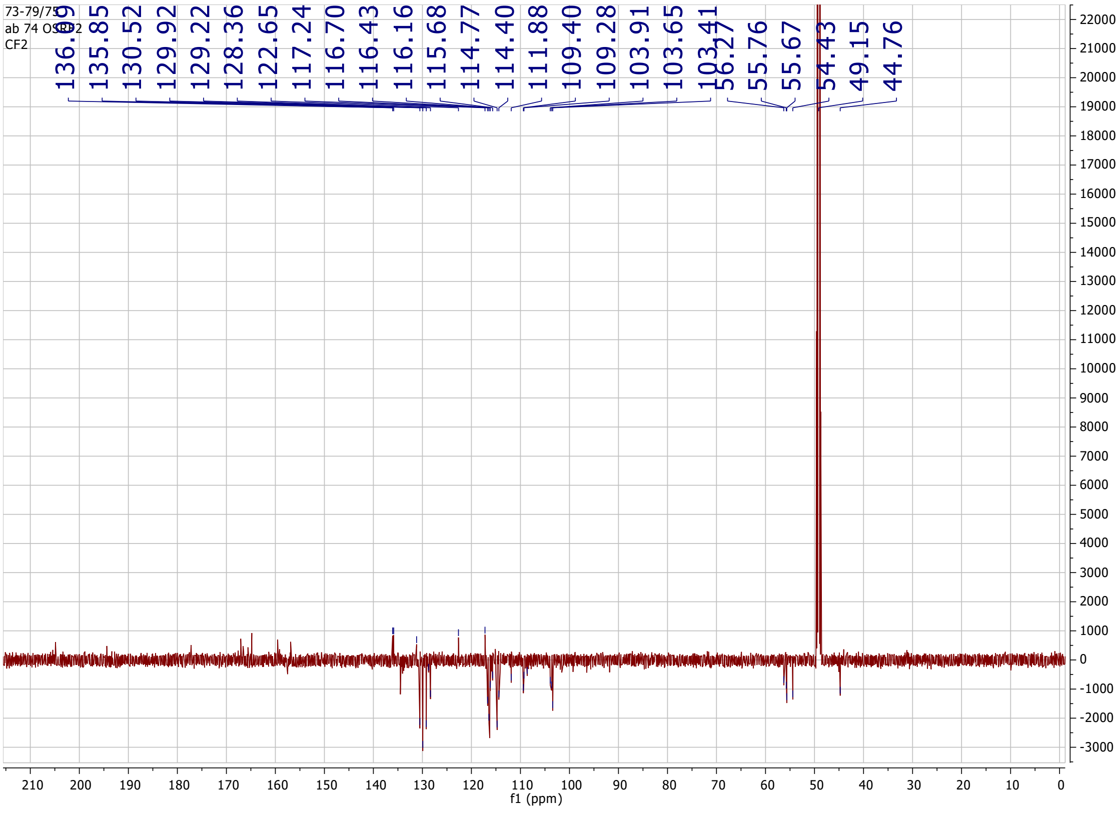

Figure S13. APT (100 MHz, MeOH-d_4) spectrum of compound **2.**

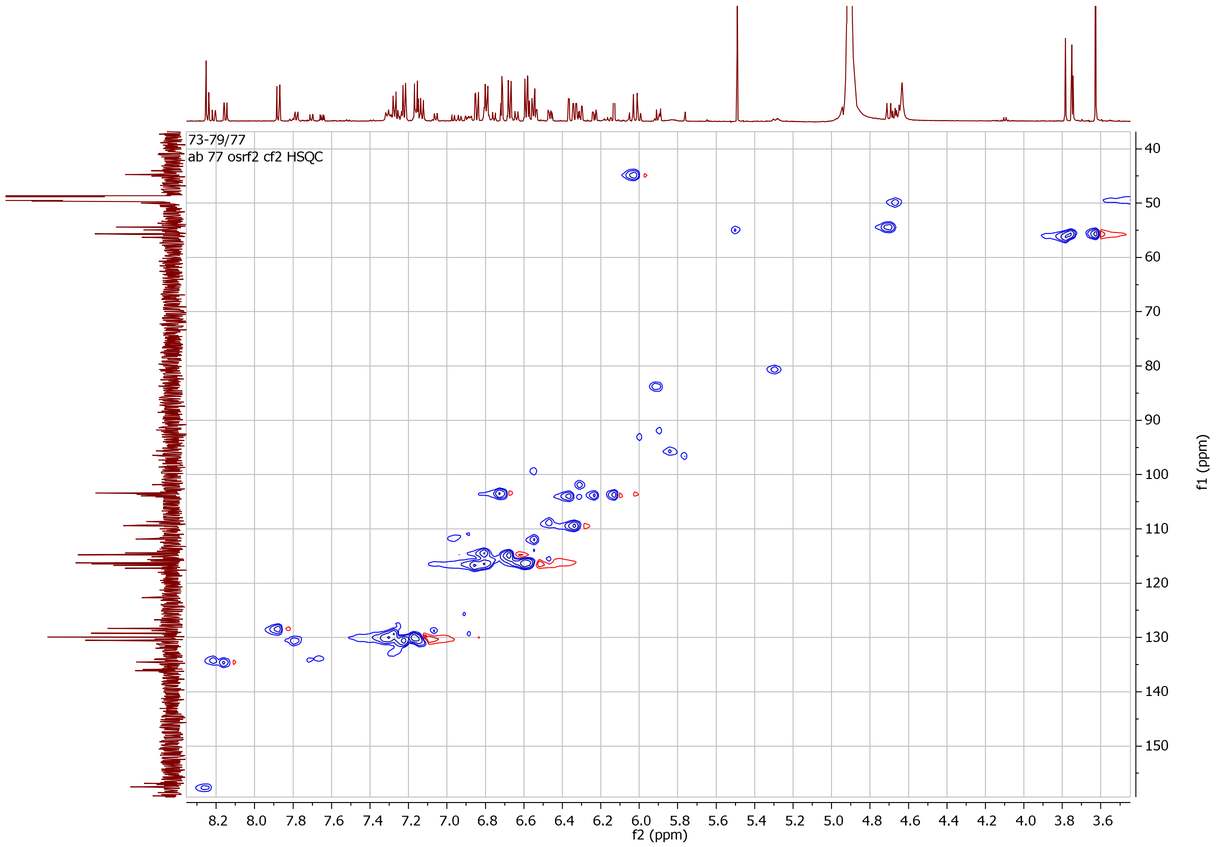

Figure S14. HSQC spectrum of compound **2.**

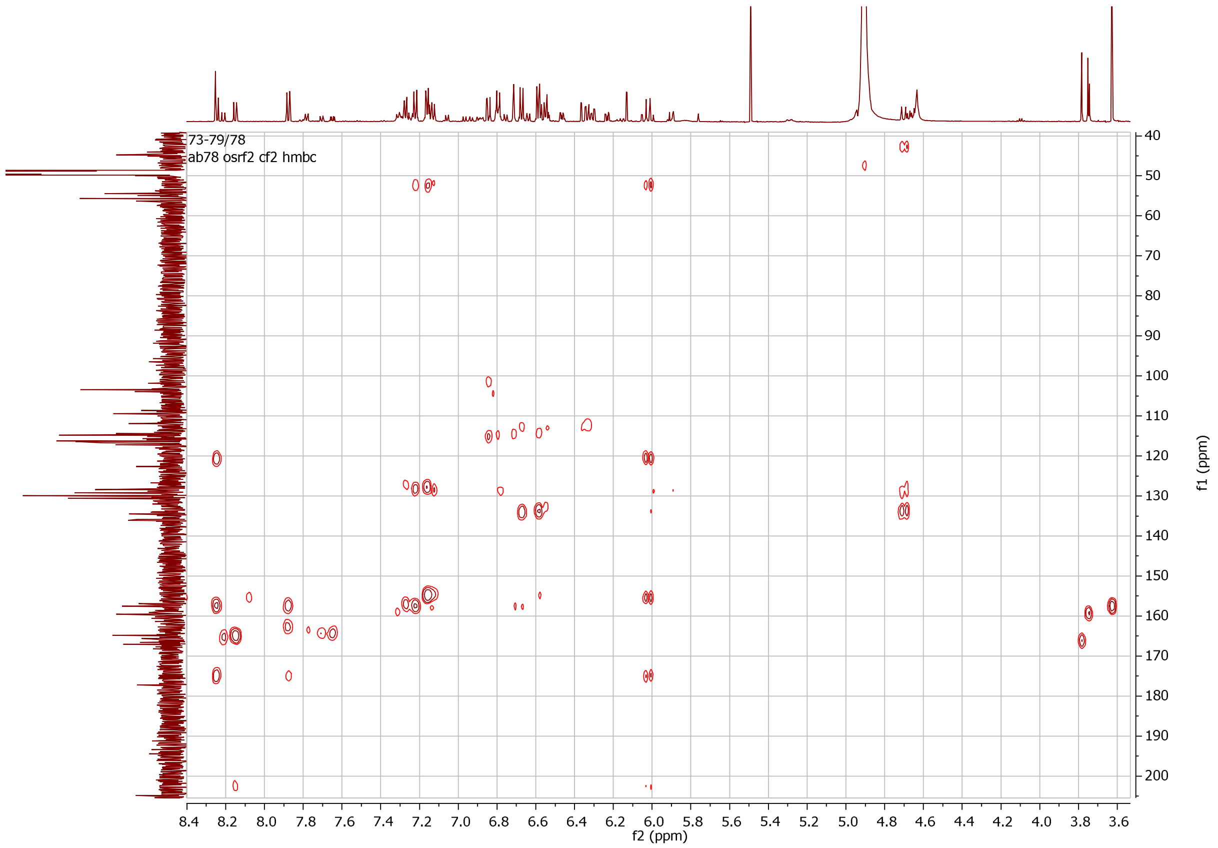

Figure S15. HMBC spectrum of compound **2.**

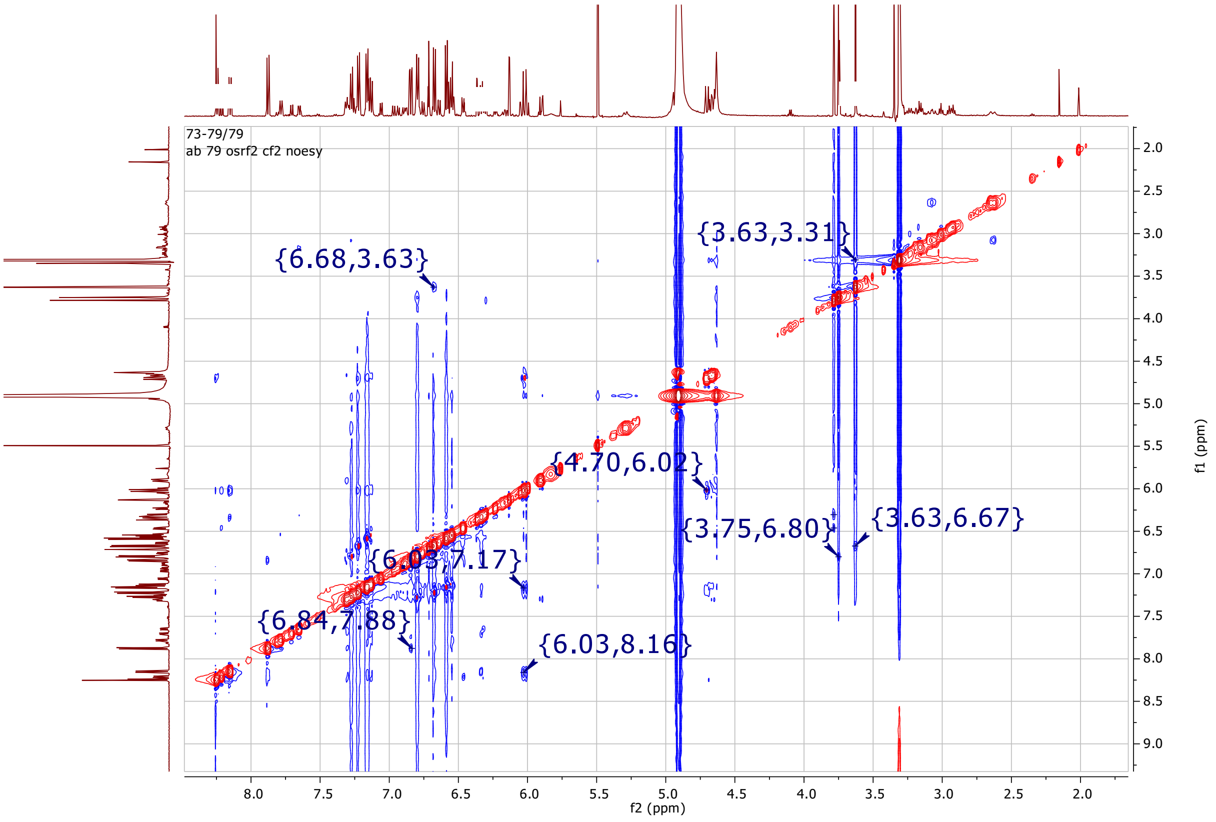

Figure S16. ROESY spectrum of compound **2.**

Compound 3

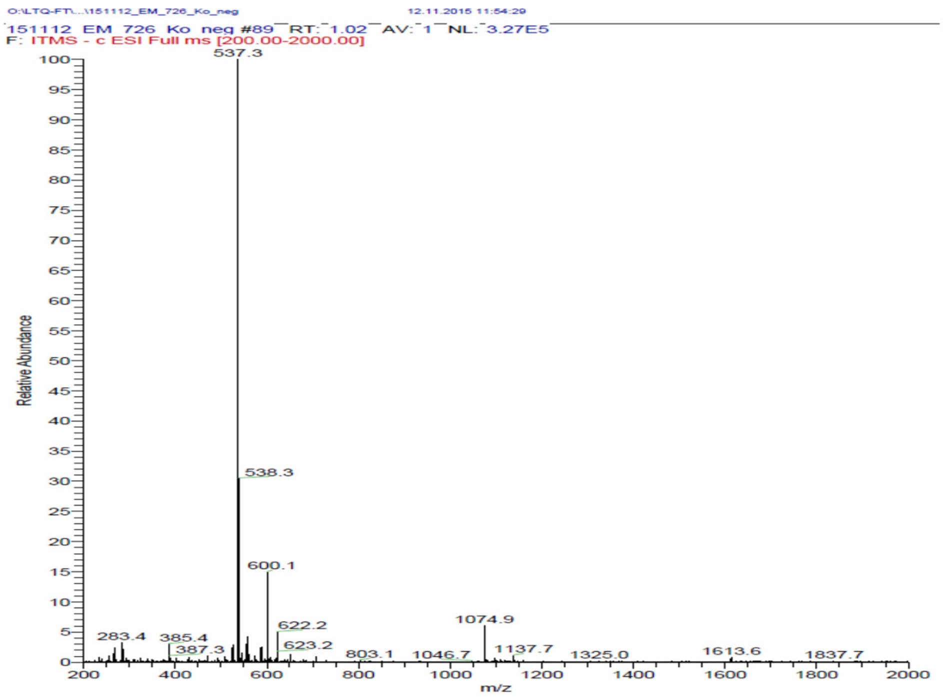

Figure S17. ESIMS Spectrum of compound **3.**

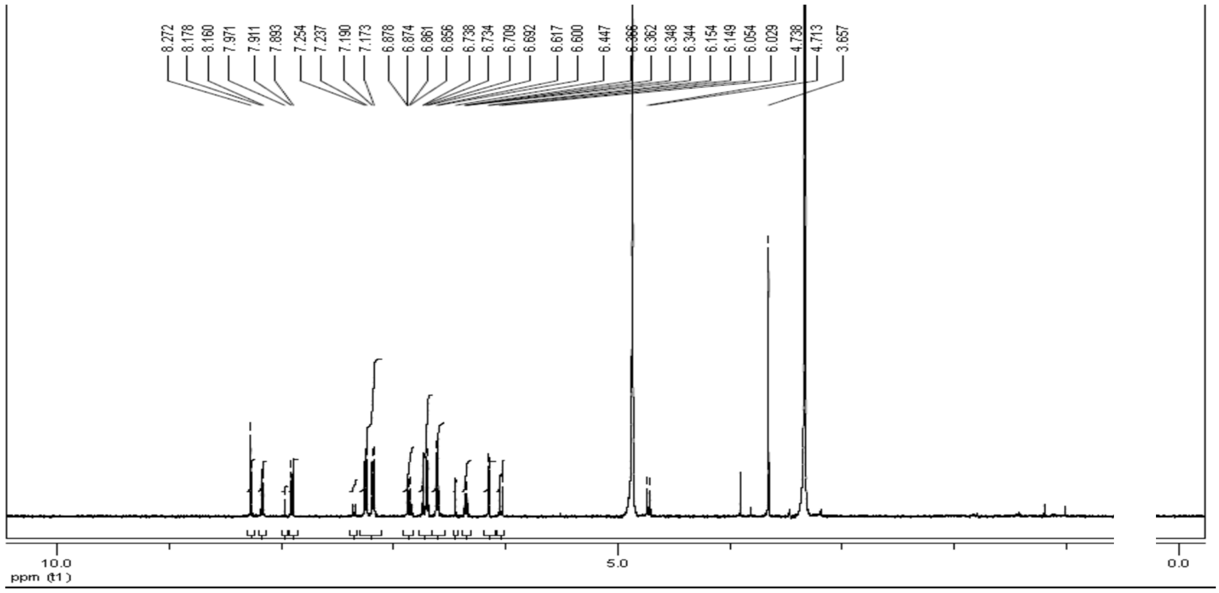

Figure S18. ^{1}H NMR (400 MHz, MeOH-d_4) spectrum of compound **3.**

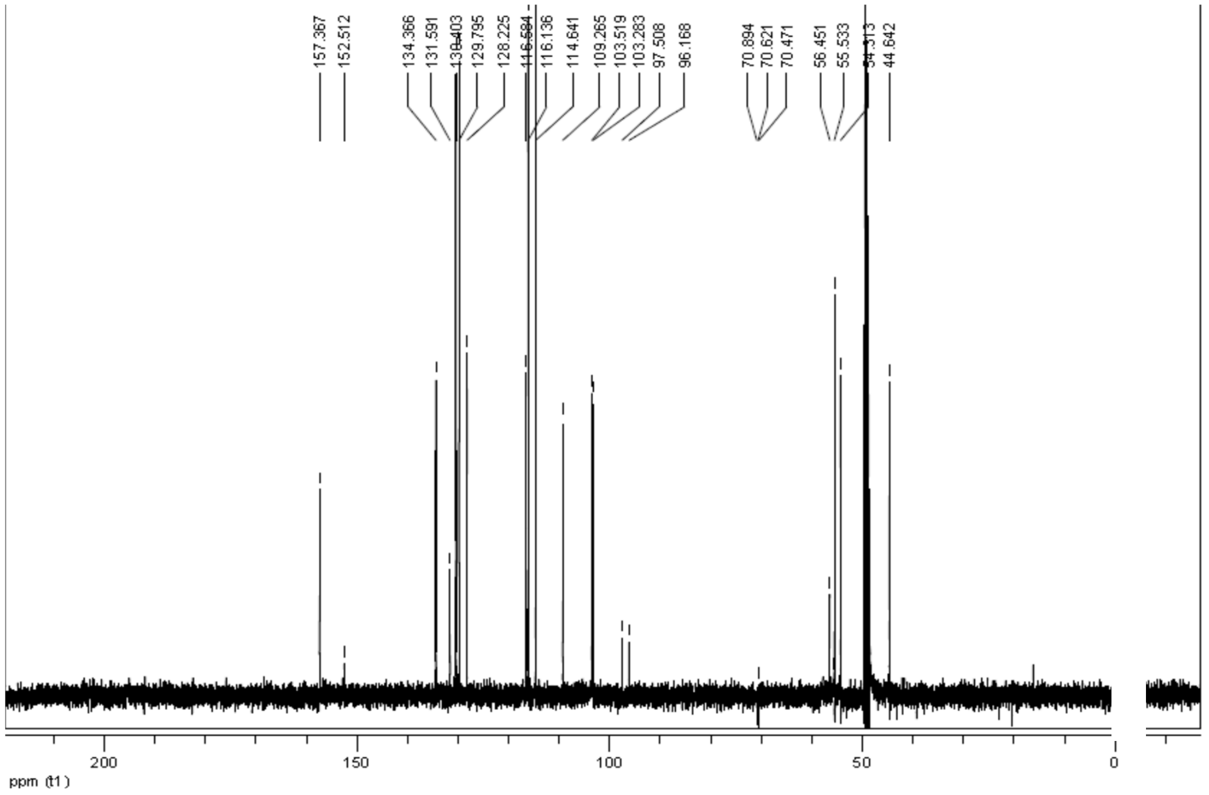

Figure S19. DEPT (100 MHZ, MeOH-d_4) spectrum of compound **3.**

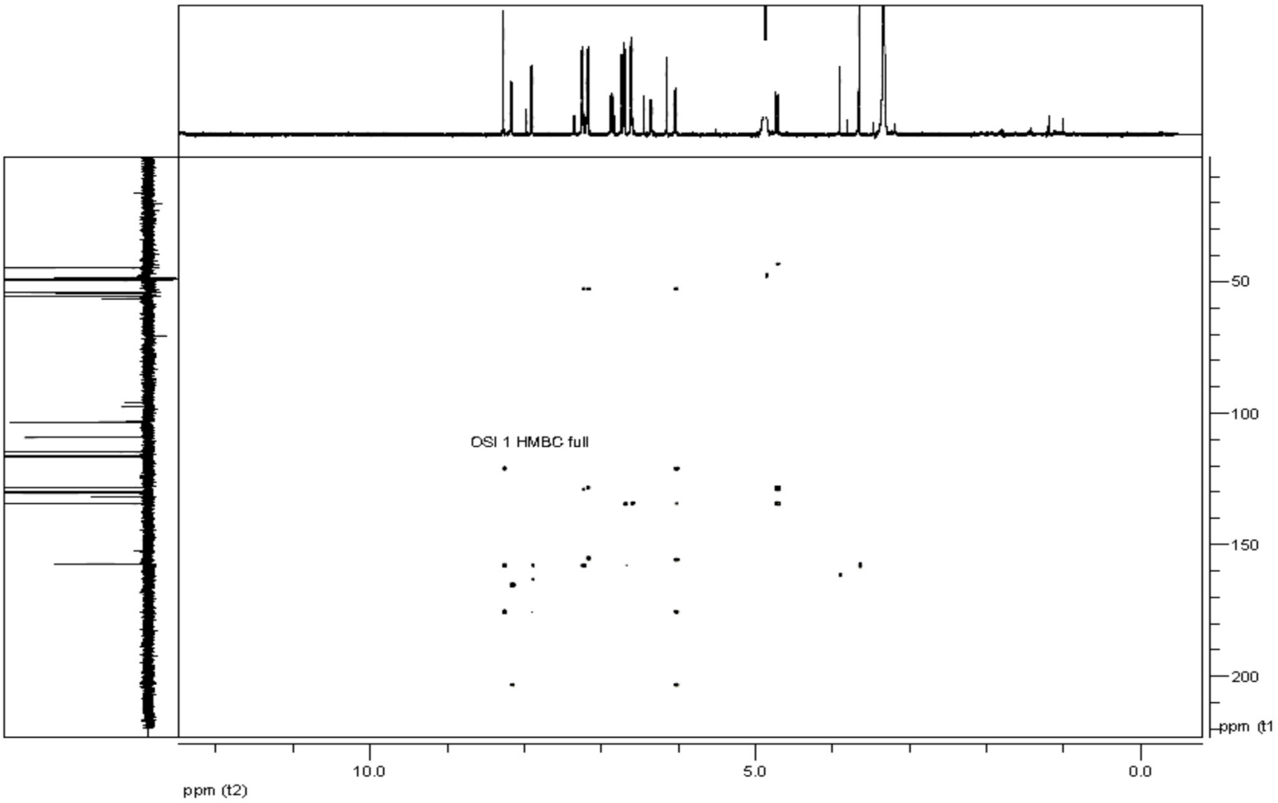

Figure S20. HMBC spectrum of compound **3.**

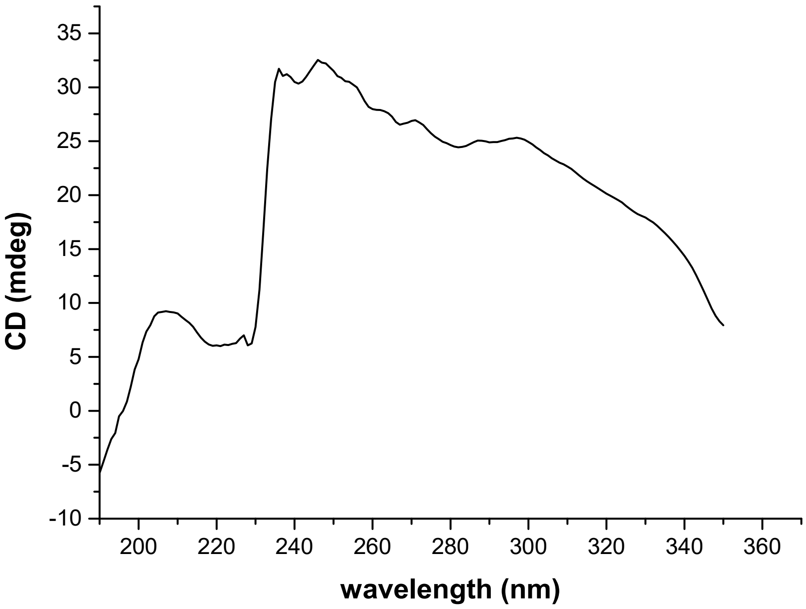

Figure S21. Experimental CD of compound **1.**

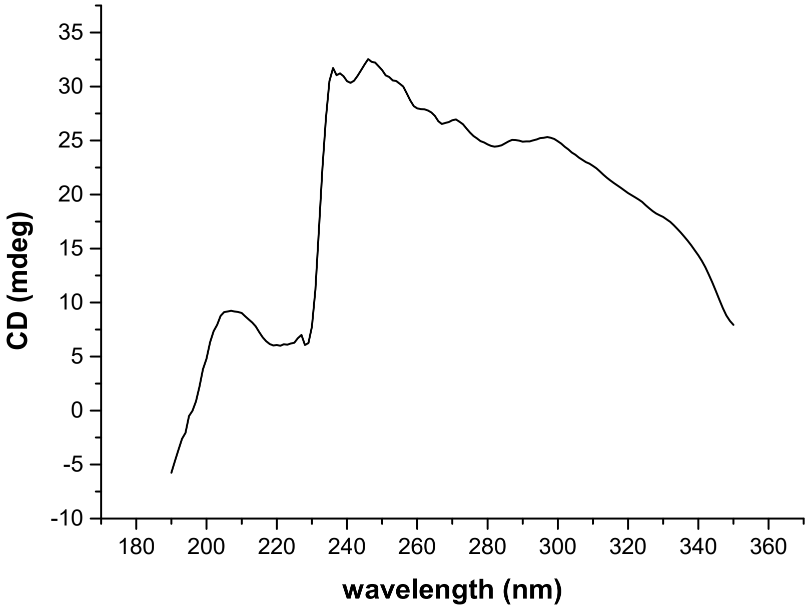

Figure S22. Experimental CD of compound **2.**
